# Design of cell-type-specific hyperstable IL-4 mimetics via modular de novo scaffolds

**DOI:** 10.1038/s41589-023-01313-6

**Published:** 2023-04-06

**Authors:** Huilin Yang, Umut Y. Ulge, Alfredo Quijano-Rubio, Zachary J. Bernstein, David R. Maestas, Jung-Ho Chun, Wentao Wang, Jian-Xin Lin, Kevin M. Jude, Srujan Singh, Brian T. Orcutt-Jahns, Peng Li, Jody Mou, Liam Chung, Yun-Huai Kuo, Yasmin H. Ali, Aaron S. Meyer, Warren L. Grayson, Nicola M. Heller, K. Christopher Garcia, Warren J. Leonard, Daniel-Adriano Silva, Jennifer H. Elisseeff, David Baker, Jamie B. Spangler

**Affiliations:** 1 Department of Chemical & Biomolecular Engineering, Johns Hopkins University, Baltimore, MD, USA.; 2 Translational Tissue Engineering Center, Johns Hopkins University School of Medicine, Baltimore, MD, USA.; 3 Department of Biochemistry and Institute for Protein Design, University of Washington, Seattle, WA, USA.; 4 Department of Bioengineering, University of Washington, Seattle, WA, USA.; 5 Department of Biomedical Engineering, Johns Hopkins University School of Medicine, Baltimore, MD, USA.; 6 Graduate Program in Biological Physics, Structure and Design, University of Washington, Seattle, WA, USA.; 7 Laboratory of Molecular Immunology and the Immunology Center, National Heart, Lung and Blood Institute, National Institutes of Health, Bethesda, MD, USA.; 8 Departments of Molecular and Cellular Physiology and Structural Biology, Stanford University School of Medicine, Stanford, CA, USA.; 9 Department of Bioengineering, University of California, Los Angeles, CA, USA.; 10 Bloomberg Kimmel Institute for Cancer Immunotherapy, Johns Hopkins University, Baltimore, MD, USA.; 11 College of Medicine, Florida State University, Tallahassee, FL, USA.; 12 Department of Bioinformatics, University of California, Los Angeles, CA, USA.; 13 Jonsson Comprehensive Cancer Center, University of California, Los Angeles, CA, USA.; 14 Eli and Edythe Broad Center of Regenerative Medicine and Stem Cell Research, University of California, Los Angeles, CA, USA.; 15 Department of Materials Science and Engineering, Johns Hopkins University, Baltimore, MD, USA.; 16 Institute for Nanobiotechnology, Johns Hopkins University, Baltimore, MD, USA.; 17 Department of Anesthesiology and Critical Care Medicine, Johns Hopkins University, School of Medicine, Baltimore, MD, USA.; 18 Allergy and Clinical Immunology, Johns Hopkins University, School of Medicine, Baltimore, MD, USA.; 19 Howard Hughes Medical Institute, Stanford University School of Medicine, Stanford, CA, USA.; 20 Howard Hughes Medical Institute, University of Washington, Seattle, WA, USA.; 21 Department of Oncology, The Johns Hopkins University School of Medicine, Baltimore, MD, USA.; 22 Sidney Kimmel Cancer Center, The Johns Hopkins University, Baltimore, MD, USA.; 23 Department of Ophthalmology, Johns Hopkins School of Medicine, Baltimore, MD, USA.; 24 These authors contributed equally: Huilin Yang, Umut Y. Ulge, Alfredo Quijano-Rubio, Zachary J. Bernstein.

## Abstract

The interleukin-4 (IL-4) cytokine plays a critical role in modulating immune homeostasis. Although there is great interest in harnessing this cytokine as a therapeutic in natural or engineered formats, the clinical potential of native IL-4 is limited by its instability and pleiotropic actions. Here, we design IL-4 cytokine mimetics (denoted Neo-4) based on a de novo engineered IL-2 mimetic scaffold and demonstrate that these cytokines can recapitulate physiological functions of IL-4 in cellular and animal models. In contrast with natural IL-4, Neo-4 is hyperstable and signals exclusively through the type I IL-4 receptor complex, providing previously inaccessible insights into differential IL-4 signaling through type I versus type II receptors. Because of their hyperstability, our computationally designed mimetics can directly incorporate into sophisticated biomaterials that require heat processing, such as three-dimensional-printed scaffolds. Neo-4 should be broadly useful for interrogating IL-4 biology, and the design workflow will inform targeted cytokine therapeutic development.

Cytokines play a central role in immunity, directing immune cell function through complex pleiotropic signaling networks. The interleukin-4 (IL-4) cytokine orchestrates both regulatory and stimulatory immune effects. Best known for its coordination of type 2 inflammatory responses by triggering the differentiation of naive T cells into T helper 2 (T_H_2) cells and suppressing type 1 inflammation, IL-4 also plays an important role in defense against parasites, promoting wound healing and mediating humoral homeostasis^1–3^. Paradoxically, IL-4 also mediates type 2 inflammation by activating a positive feedback loop in T_H_2 cells that increases production of IL-4 and other type 2 cytokines, which drives immunoglobulin class switching in B cells, promotes eosinophil transmigration and increases mucus secretion^2,3^. These proinflammatory effects of IL-4 can lead to development and pathogenesis of allergies and asthma^2,3^.

IL-4 signals through two different heterodimeric receptor complexes composed of either the IL-4 receptor-α (IL-4Rα) and common γ (γ_c_) chains (type I complex) or the IL-4Rα and IL-13Rα1 chains (type II complex)^1^. IL-4 binds its private IL-4Rα chain with high affinity (*K*_d_ < 1 nM)^4^, and the IL-4–IL-4Rα complex can bind to either γ_c_ or IL-13Rα1 to form type I or type II complexes with *K*_d_ values of 559 nM and 487 nM, respectively^4^. The type I complex is typically expressed on hematopoietic cells, whereas the type II complex is predominantly expressed on non-hematopoietic cells^4–6^. IL-4 stimulates cellular signaling pathways through both complexes, principally through phosphorylation of signal transducer and activator of transcription 6 (STAT6)^7,8^. The type II complex is shared between IL-4 and IL-13, although unlike IL-4, IL-13 cannot signal through the type I complex^4^. By engaging with a common set of receptor chains, IL-4 and IL-13 exhibit similarities in their activities, especially in mediating type 2 inflammation. While type I receptor engagement activates the insulin receptor substrate signaling cascade more potently than type II engagement^9^, type I versus type II signaling effects have been difficult to decouple because no natural cytokine exclusively signals through the type I complex. Evidence suggests that IL-4 and IL-13 have similar yet distinctive immune functions, and understanding their receptor signaling activities will enable elucidation of their respective roles^2,3^.

IL-4 has been explored as a potential therapeutic agent to redirect T and B cell activities, but toxicities related to its pleotropic functions have limited its use in the clinic^3,10,11^. To better regulate the effects of IL-4, investigators have designed therapeutically relevant molecules targeting the IL-4 pathway, including several monoclonal antibodies to IL-4 or IL-4 receptor chains^12^ and engineered variants of the IL-4 cytokine^5,11,13,14^. The low stability of the native cytokine structure^15^, potential immunogenicity toward natural cytokine variants^16^ and the complexity of cytokine–receptor interfaces complicate the development of therapeutics derived from natural cytokines, such as IL-4. One alternative protein engineering route is de novo computational design of proteins. In contrast with natural cytokines, de novo designed proteins have highly stable structures that resist thermal denaturation at extreme temperatures and are robust to surface amino acid mutations^17,18^. In this way, de novo proteins allow for rational control of molecular properties, including structure, stability, immunogenicity and activity.

While early work in de novo protein design focused on creating new but inert structures, recent computational advances have enabled de novo design of functional proteins^19^. For example, the Rosetta de novo design suite has been used to create cytokine mimetics that modulate receptor dimerization^20^, protein sensors that detect binding to small molecules^21^ and protein units that form logic gates^22^. We previously reported a de novo designed hyperstable mimetic of the IL-2 cytokine, denoted neoleukin-2/15 (hereafter Neo-2)^17^. This protein mimics the functions of IL-2 and IL-15 and, because Neo-2 signals independently of the IL-2Rα chain (only engaging the IL-2Rβ and γ_c_ chains), it exhibits potent antitumor activity without eliciting systemic toxicities that are typically associated with IL-2 therapy.

This study built on the Neo-2 scaffold to design de novo mimetics of human and mouse IL-4 (hNeo-4 and mNeo-4, respectively). These proteins were created by introducing substitutions from IL-4 into Neo-2 at the IL-4Rα interface and enhancing receptor binding through affinity maturation. Both mimetics largely recapitulated the signaling and downstream biological functions of natural IL-4 without any IL-2 bioactivity. However, unlike IL-4, they signal strictly through the type I complex, offering insights into the intertwined functions of the type I and type II complexes. Moreover, due to its thermal stability, hNeo-4 could be used directly to produce biomaterials via three-dimensional (3D) printing.

## Results

### Design of IL-4 mimetics based on Neo-2 structure

**Extended Data Fig. 1 |:**
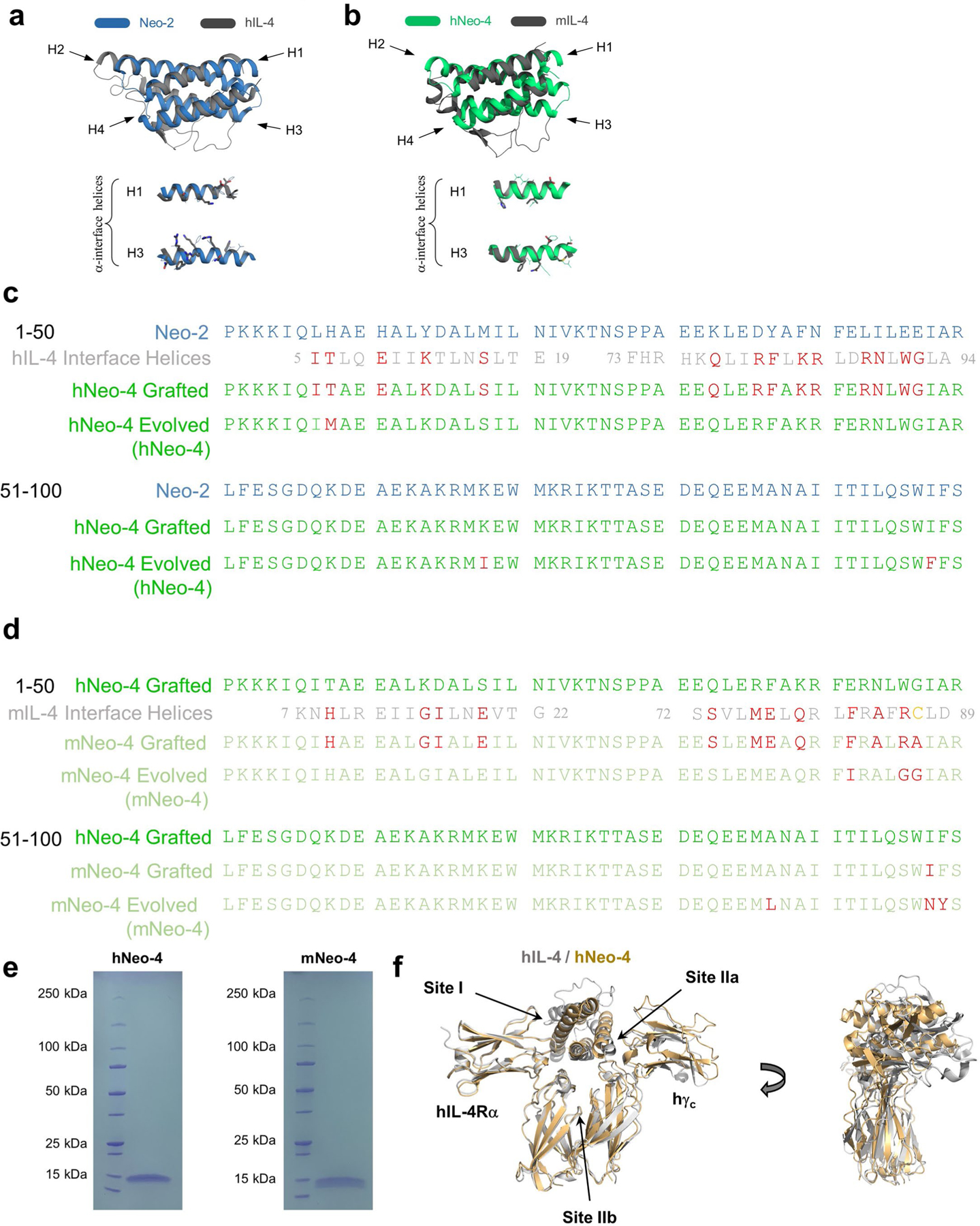
De novo design and production of Neo-4 molecules. **a**, Structural alignment of Neo-2 and hIL-4. Residues in hIL-4 at the hIL-4/hIL-4Rα interface that were grafted onto Neo-2 are depicted with sticks, and the corresponding residues on Neo-2 are depicted with lines. **b**, structural alignment of hNeo-4 and mIL-4. Residues in mIL-4 at the mIL-4/mIL-4Rα interface that were grafted onto hNeo-4 are depicted with sticks, and the corresponding residues on hNeo-4 are depicted with lines. **c**, Amino acid sequence alignment of Neo-2, designed hNeo-4, and the final evolved hNeo-4 (henceforth denoted hNeo-4), demonstrating the evolution of the hNeo-4 sequence during the development process. Mutations introduced at each round of evolution are marked in red. **d**, Amino acid sequence alignment of designed hNeo-4, designed mNeo-4 and the final evolved mNeo-4 (henceforth denoted mNeo-4), demonstrating the evolution of the mNeo-4 sequence during the development process. Mutations introduced at each round of evolution are marked in red. mIL-4 cysteine at position 87, depicted in yellow, was not grafted to the hNeo-4 structure. **e**. SDS-PAGE analysis of purified hNeo-4 and mNeo-4. Experiment was repeated once independently with similar results. **f**, AlphaFold2 predictions of the structure of hNeo-4 bound to the human type I IL-4 receptor complex (peach) aligned to the IL-4Rα subunit in the experimentally determined crystal structure of the type I hIL-4 complex (gray; PDB ID 3BPL). hNeo-4 is predicted to preserve all three binding interfaces present in the hIL-4 signaling complex.

**Extended Data Fig. 2 |:**
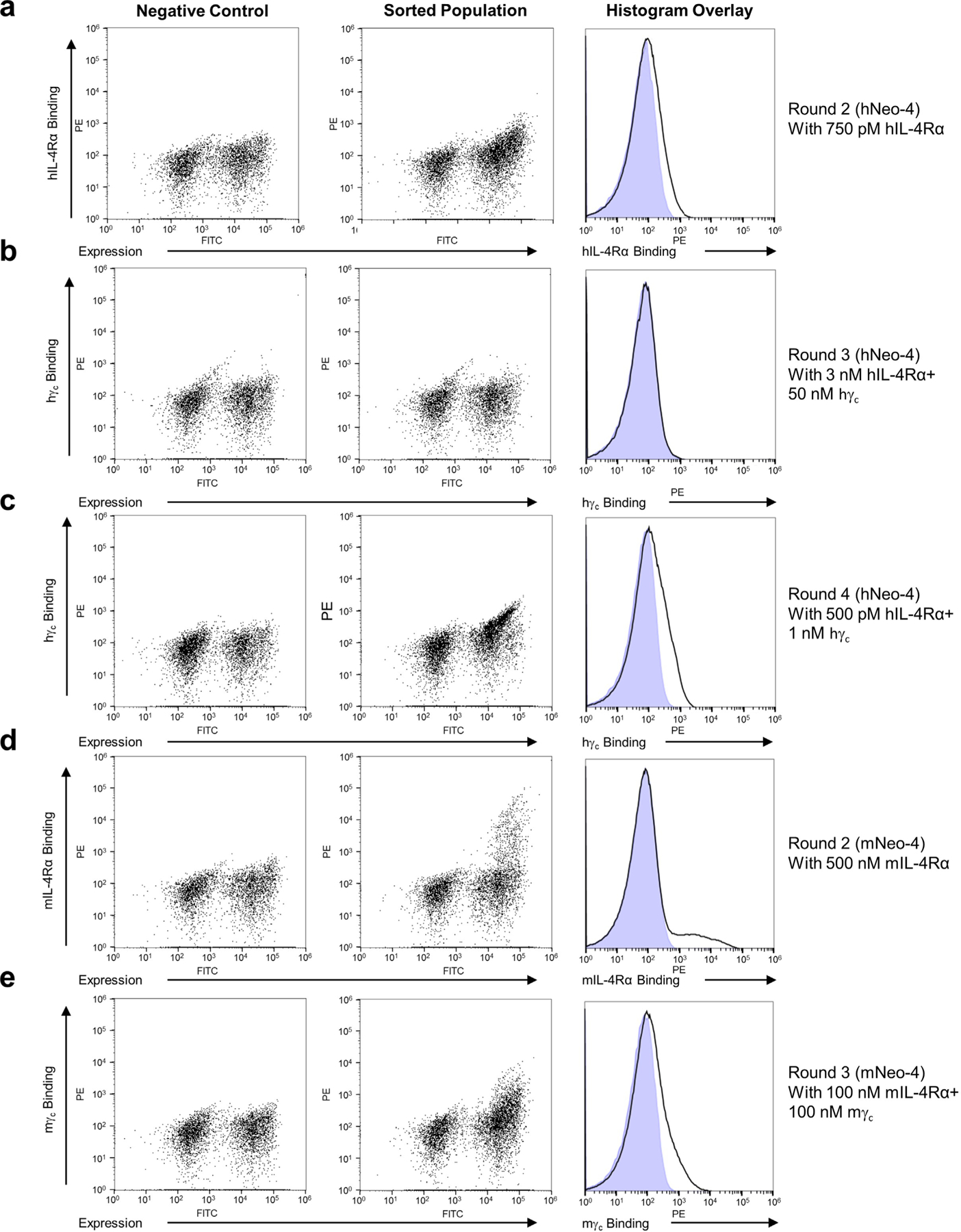
Flow cytometry plots depicting the selection process for evolution of IL-4 mimetics. Yeast libraries were incubated with fluorescent streptavidin only (Negative Control, left column) or with the indicated biotinylated receptor subunits followed by fluorescent streptavidin (Sorted Population, center column), with difference histogram shown on the right (blue represents Negative Control and line represents Sorted Population). Concentrations of soluble receptor chain(s) used in each round of selection are indicated next to the histograms. Expression of IL-4 mimetics on the yeast surface is tracked by Fluorescein isothiocyanate (FITC)-labeled anti-cmyc tag antibody and binding is quantified by phycoerythrin (PE)-labeled streptavidin. **a-c**, Rounds 2, 3, and 4 of human IL-4 mimetic evolution. **d-e**, Rounds 2 and 3 of mouse IL-4 mimetic evolution.

**Fig. 1 |:**
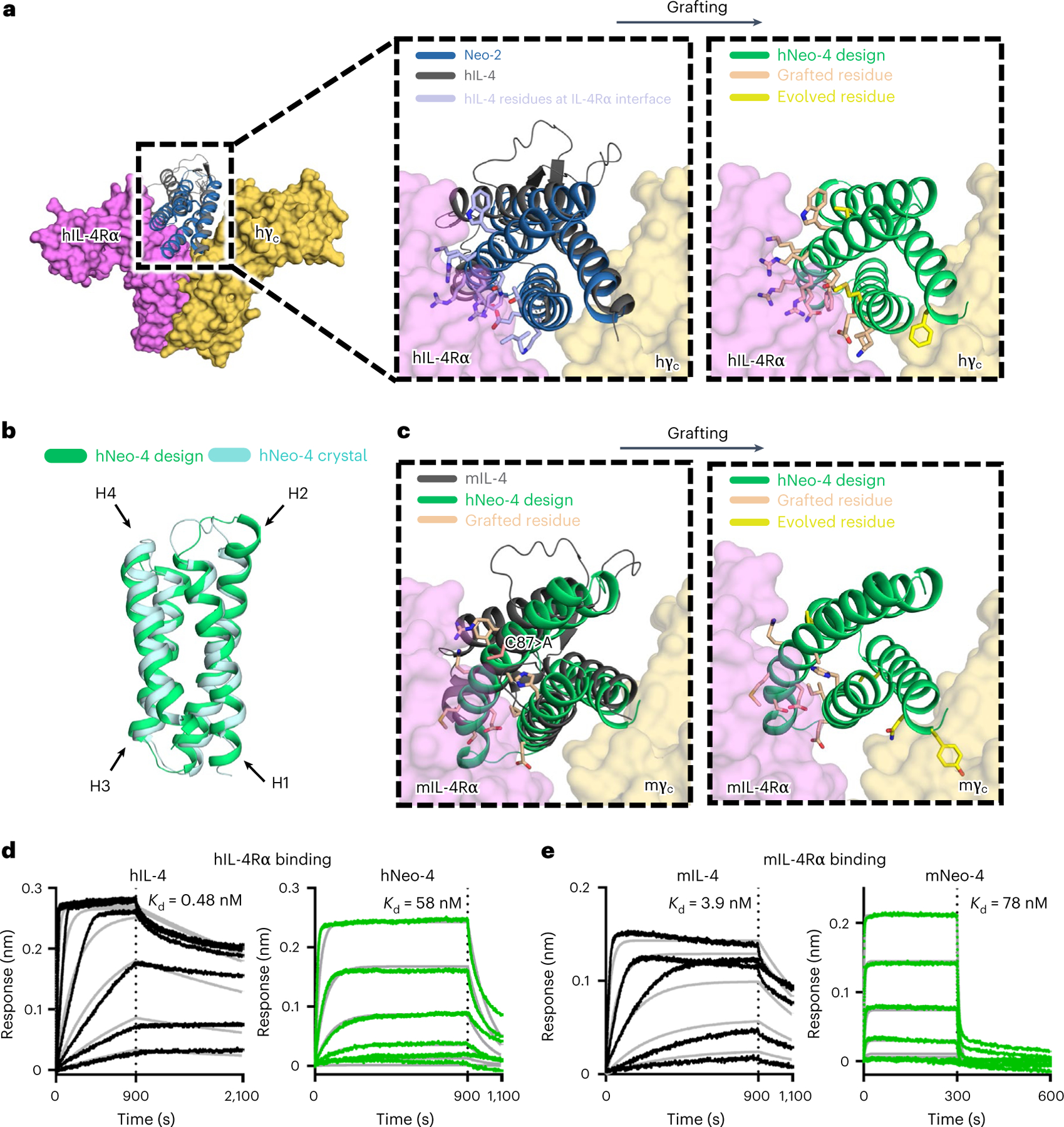
Computational design of IL-4 mimetics. **a**, Progression of the hNeo-4 evolution process. hIL-4 residues at the hIL-4–hIL-4Rα interface were grafted onto the Neo-2 structure, and the resulting molecule was subjected to directed evolution to improve its binding affinity toward hIL-4 receptors. **b**, Comparison of the computationally predicted hNeo-4 structure and the experimentally determined hNeo-4 crystal structure. **c**, Progression of the mNeo-4 evolution process. mIL-4 residues at the mIL-4–mIL-4Rα interface were grafted onto the designed hNeo-4 structure, and the resulting molecule was subjected to directed evolution to improve its binding affinity toward mIL-4 receptors. Cys 87 in mIL-4 at the mIL-4–mIL-4Rα interface, depicted in salmon, was not grafted onto the hNeo-4 structure to avoid an unpaired cysteine bridge. Instead, alanine was substituted into the corresponding position 47 in hNeo-4 before directed evolution. **d**,**e**, BLI sensograms depicting the interactions between hIL-4 or hNeo-4 (threefold serial dilutions starting at 200 nM for both) and hIL-4Rα (**d**) and between mIL-4 or mNeo-4 (threefold serial dilutions starting at 67 nM for mIL-4 or starting at 260 nM for mNeo-4) and mIL-4Rα (**e**). *K*_d_ values derived from the kinetic parameters are indicated. Raw data were fitted using a 1:1 Langmuir binding model. Fitted curves are shown in gray.

Despite their distinct functions, the natural hIL-2 and hIL-4 cytokines both have classic four-helix-bundle structures and belong to the γ_c_ cytokine family based on sharing this receptor chain^1^. Capitalizing on commonalities between these two molecules, we used our de novo IL-2 mimetic (Neo-2)^17^ as a scaffold for the design of IL-4 mimetics. We first grafted 14 cytokine residues implicated in the hIL-4–hIL-4Rα interface onto Neo-2 (Fig. 1a, Extended Data Fig. 1a,c and Supplementary Table 1), while preserving Neo-2 residues implicated in the Neo-2–hγ_c_ interface. The resulting grafted hNeo-4 was then subjected to random mutagenesis and four rounds of selection using the yeast surface display platform to increase hIL-4Rα binding (Extended Data Fig. 2a–c and Supplementary Table 3). Clones from the converged library were expressed in *Escherichia coli*, and the molecule with the highest binding affinity for hIL-4Rα was selected (hereafter hNeo-4; Extended Data Fig. 1e and Supplementary Table 4). Overall, 16 mutations were introduced into Neo-2 to derive hNeo-4; 1 of the 14 originally grafted mutations was modified by selection, and 2 more mutations were introduced during selection. The molecular structure of hNeo-4 was determined via X-ray crystallography to a resolution of 2.97 Å and showed atomic agreement with the computationally predicted model (Fig. 1b). The topology of hNeo-4 in complex with the hIL-4 type I complex was predicted using AlphaFold2, which showed the same overall topology as the native type I hIL-4 cytokine–receptor complex (Extended Data Fig. 1f). We were unable to predict the mNeo-4–mIL-4Rα–mγ_c_ complex structure, presumably because the measured low affinity of the complex reflects a low level of complementarity at site IIa on mγ_c_.

Unlike hIL-2, which cross-reacts with the mIL-2 receptor subunits^23^, hIL-4 does not cross-react with the mIL-4 receptor chains^24^. Therefore, to characterize computationally designed IL-4 mimetics in vivo, we designed a version of hNeo-4 that reacts with mouse receptors (denoted mNeo-4). We speculated that differential disulfide bridges in hIL-4 (between helices 1 and 4) and mIL-4 (between helices 1 and 3) may contribute to the lack of cross-reactivity for the natural cytokine. Because hNeo-4 has no disulfide bridges, we anticipated that a mouse mimic could be generated by grafting the mIL-4 interface with mIL-4Rα onto hNeo-4. We therefore grafted 12 mIL-4 residues implicated in the mIL-4Rα interface onto hNeo-4 (Supplementary Table 2). In addition, because the mIL-4 interface includes a cysteine residue (Cys 87) that participates in a disulfide bridge that was not reproduced in the mimetic, this residue was grafted as an alanine. The resulting design was subjected to random mutagenesis and enriched for mIL-4Rα binding via yeast surface display-mediated directed evolution (Fig. 1c, Extended Data Figs. 1b,d and 2d,e and Supplementary Table 3). The highest-affinity clone, termed mNeo-4, was expressed and purified using *E. coli* (Extended Data Fig. 1e and Supplementary Table 4). This clone contained 15 mutations relative to hNeo-4; 3 of the 12 originally grafted mutations were modified by selection, and 3 more were introduced during selection.

**Extended Data Fig. 3 |:**
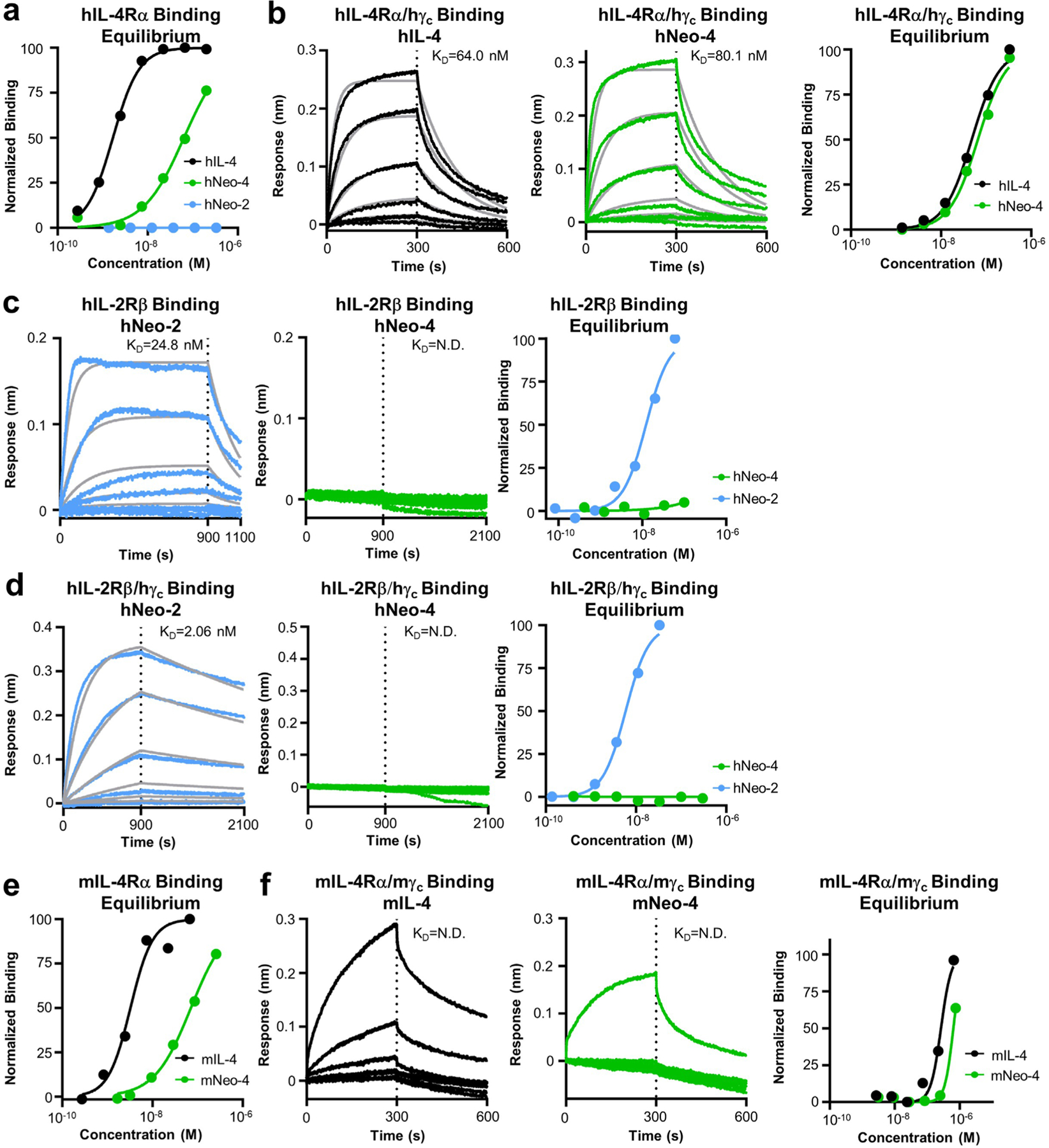
Characterization of Neo-4 binding properties. **a**, Equilibrium bio-layer interferometry (BLI) titrations of hIL-4 or hNeo-4 against immobilized hIL-4Rα. **b**, BLI sensograms depicting interactions between hIL-4 or hNeo-4 and the hIL-4Rα/hγ_c_ complex. Equilibrium dissociation constants (K_D_) derived from the kinetic parameters are indicated. Equilibrium BLI titrations of hIL-4 or hNeo-4 (3-fold serial dilutions starting at 333 nM for both) against the hIL-4Rα/hγ_c_ complex are shown at *right*. **c-d**, BLI sensograms depicting the interaction between hNeo-4 or Neo-2 and immobilized (**c**) IL-2Rβ and (**d**) hIL-2Rβ/hγ_c_ complex. In (**c**) biosensors were exposed to 3-fold serial dilutions of Neo-2 starting at 60 nM or hNeo-4 starting at 100 nM, respectively; and in (**d**) biosensors were exposed to 3-fold serial dilutions of Neo-2 starting at 33 nM or hNeo-4 starting at 300 nM, respectively. K_D_ values derived from the kinetic parameters are indicated. Equilibrium BLI titrations of hNeo-4 or Neo-2 against immobilized (**c**) hIL-2Rβ and (**d**) hIL-2Rβ/hγ_c_ are shown at *right*. **e**, Equilibrium BLI titrations of mIL-4 or mNeo-4 against immobilized mIL-4Rα extracellular domain. **f**, BLI sensograms depicting interactions between mIL-4 or mNeo-4 (3-fold serial dilutions starting at 650 nM for mIL-4 and 750 nM for mNeo-4) and mIL-4Rα/mγ_c_ complexes. K_D_ values derived from the kinetic parameters are indicated. Equilibrium BLI titrations of mIL-4 or mNeo-4 against the mIL-4Rα/mγ_c_ complex are shown at *right*. All raw data were fitted using a 1:1 Langmuir binding model. Fitted curves are shown in gray.

To compare the biophysical properties of engineered and natural IL-4 cytokines, we evaluated the binding affinities of hNeo-4 and hIL-4 for both hIL-4Rα and the hIL-4Rα–hγ_c_ complex via biolayer interferometry (BLI; Fig. 1d, Extended Data Fig. 3a,b and Supplementary Table 5). Similar to previous reports^4^, *K*_d_ values for hIL-4 binding to hIL-4Rα and the hIL-4Rα–hγ_c_ complex were 0.48 nM and 64 nM, respectively. hNeo-4 had a 121-fold weaker binding affinity toward hIL-4Rα (*K*_d_ = 58 nM) than hIL-4 due to a higher rate of dissociation (*k*_off_) and a slightly lower rate of association (*k*_on_). Interestingly, hNeo-4 had comparable affinity (*K*_d_ = 80 nM) for the hIL-4Rα–hγ_c_ complex to hIL-4 (Extended Data Fig. 3b and Supplementary Table 5). We validated that the Neo-2-derived hNeo-4 did not exhibit residual interactions with hIL-2Rβ or the hIL-2Rβ–hγ_c_ complex (Extended Data Fig. 3c,d), confirming that the specificity was fully transformed. We also conducted BLI studies on mIL-4 and mNeo-4 (Fig. 1e, Extended Data Fig. 3e,f and Supplementary Table 5). mNeo-4 exhibited 20-fold weaker binding affinity for mIL-4Rα (*K*_d_ = 78 nM) than mIL-4 (*K*_d_ = 3.9 nM). mNeo-4 also showed weaker binding to the mIL-4Rα–mγ_c_ complex than mIL-4, although binding parameters were outside the range of instrument sensitivity. The homology between hNeo-4 and mNeo-4 is 85%, whereas that of natural hIL-4 and mIL-4 is only 40%; thus, without dramatically altering the protein sequence, we engineered cytokine mimetics for different species, highlighting the flexibility of the de novo design platform.

### IL-4 mimetics recapitulate cellular activity of native IL-4

**Extended Data Fig. 4 |:**
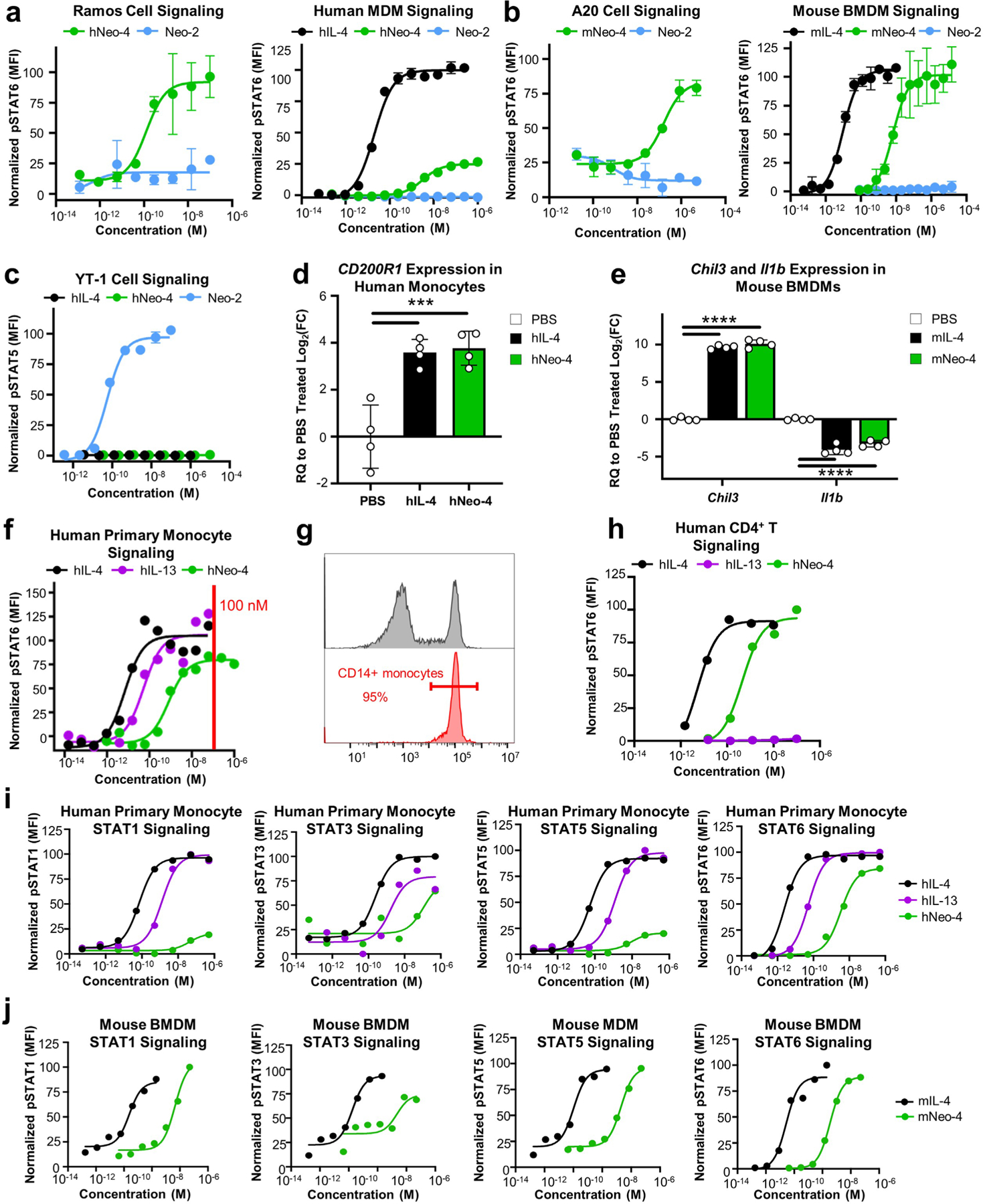
Characterization of Neo-4 bioactivity in vitro. **a**, STAT6 phosphorylation induced by hIL-4, hNeo-4, or Neo-2 in human Ramos B cells (*n* = 3) and primary monocyte-derived macrophages (MDMs) (*n* = 3). **b**, STAT6 phosphorylation induced by mIL-4, mNeo-4, or Neo-2 in mouse A20 B cells (*n* = 3) and primary bone marrow-derived macrophages (BMDMs) (*n* = 4) **c**, STAT5 phosphorylation induced by hIL-4, hNeo-4, or Neo-2 in human YT-1 natural killer cells (*n* = 3). Data represent mean ± SD. **d**, qRT-PCR analysis of *CD200r1* expression in primary human monocytes treated with hIL-4 or hNeo-4 (*n* = 4). Data represent mean ± SD (*n* = 4). ^****^p < 0.0001, one-way ANOVA. **e**, *Chil3, Il1b* expression in primary mouse BMDMs treated with mIL-4 and mNeo-4. Data represent mean ± SD (*n* = 4 biologically independent samples). ^****^p < 0.0001, two-way ANOVA. **f**, STAT6 phosphorylation induced by hIL-4, hIL-13, or hNeo-4. In human primary monocytes. Saturating concentrations of all 3 protein treatments were determined to be 100 nM, as indicated by the red line, guiding dosing for RNA-seq studies. Data represent mean ± SD (*n* = 2). **g**, Flow cytometry histograms showing CD14 expression levels on PBMCs before (gray) and after (red) CD14^+^ cell sorting. The sorted cells were considered monocytes. For ease of visualization, only significance compared to the control cohort is shown in panels **d-e**. All p-values are recorded in Supplementary Table 7. **h**, STAT6 phosphorylation induced by hIL-4, hIL-13, or hNeo-4 in human CD4^+^ T cells. **i**, Phosphorylation of STAT1, STAT3, and STAT5 induced by hIL-4, hIL-13, or hNeo-4 in human primary monocytes. Data represent mean ± SD (*n* = 2). **j**, Phosphorylation of STAT1, STAT3, and STAT5 induced by mIL-4 or mNeo-4 in mouse BMDMs. Data represent mean ± SD (*n* = 2).

**Fig. 2 |:**
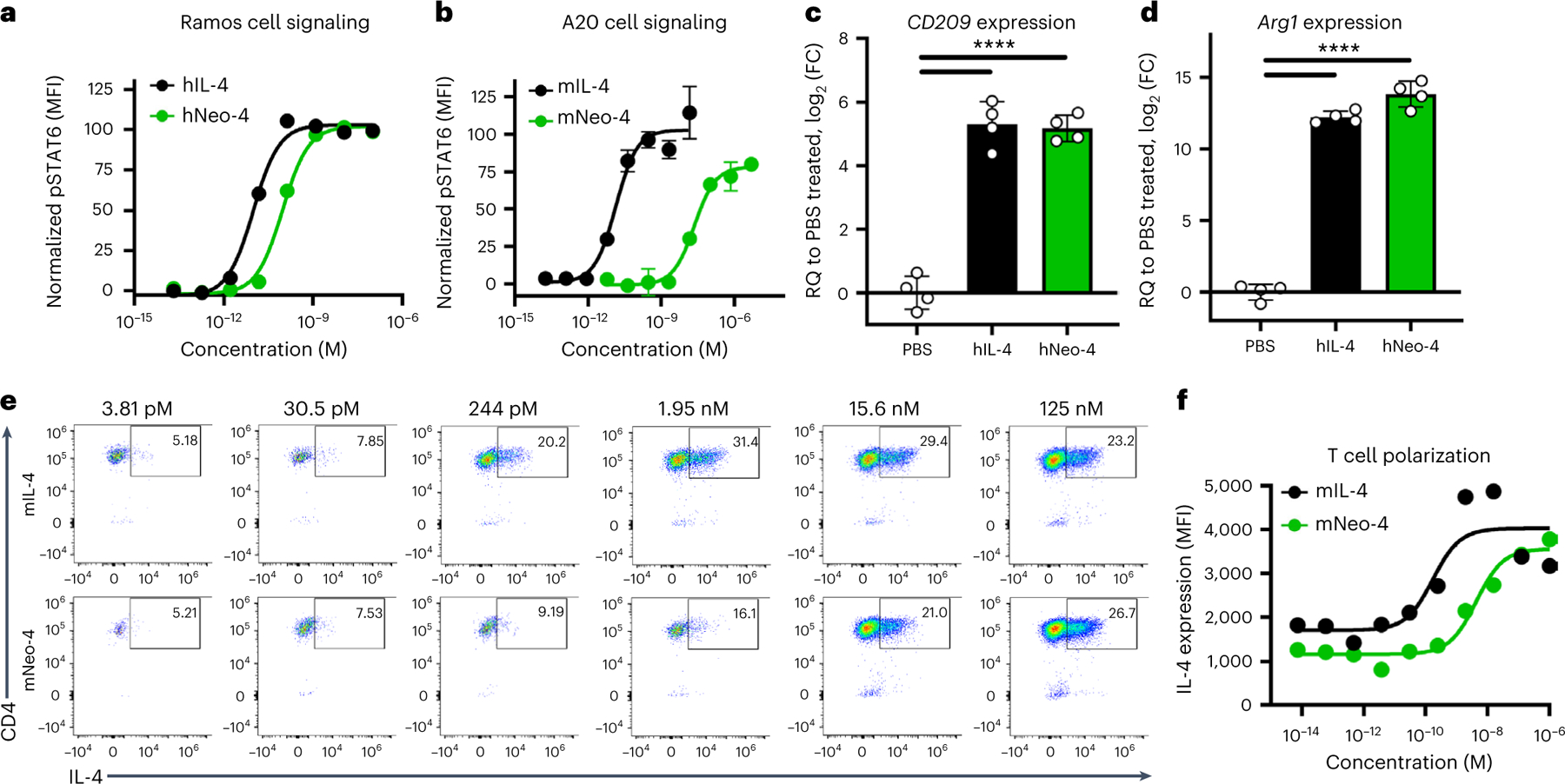
IL-4 mimetics recapitulate functions of the natural IL-4 cytokine in vitro. **a**,**b**, STAT6 phosphorylation induced by hIL-4 and hNeo-4 in Ramos B cells (**a**) or mIL-4 and mNeo-4 in mouse A20 cells (**b**), as measured by flow cytometry. Data represent mean ± s.d. (*n* = 3 biologically independent samples). **c**,**d**, RT–qPCR analysis of *CD209* expression in human primary monocytes treated with hIL-4 or hNeo-4 (**c**) and *Arg1* expression in mouse BMDMs treated with mIL-4 or mNeo-4 (**d**). Data represent mean ± s.d. (*n* = 4 biologically independent samples); ****P < 0.0001. Data were analyzed by one-way analysis of variance (ANOVA); RQ, relative quantification. **e**, Flow cytometry plots demonstrating stimulation of mIL-4–enhanced green fluorescent protein (mIL-4–eGFP) expression in primary naive T cells from 4GET mice in a dose-dependent fashion following incubation with mIL-4 or mNeo-4. **f**, Dose–response curves showing increased mean fluorescence intensity (MFI) of induced mIL-4–eGFP expression following stimulation with mIL-4 and mNeo-4 (*n* = 1). For ease of visualization, only significance compared to the control cohort is shown in **c** and **d**. All *P* values are provided in Supplementary Table 7.

Using IL-4-induced STAT6 phosphorylation as a readout, we interrogated the activity of our mimetics on different IL-4-sensitive human and mouse cell lines compared to the natural cytokines (Fig. 2a,b and Extended Data Fig. 4a,b). On Ramos human B cells, which express predominantly type I receptors, hIL-4 and hNeo-4 potently activated STAT6 with half-maximal effective concentrations (EC_50_) of 11 pM and 140 pM, respectively (Fig. 2a), consistent with the discrepancy in their binding affinities to hIL-4Rα. Interestingly, although the affinities of hNeo-4 and hIL-4 for the hIL-4Rα–hγ_c_ complex are similar (Supplementary Table 5), hNeo-4 is greater than tenfold less potent than hNeo-4 with respect to cell signaling. This can be attributed to binding differences in BLI versus in live cells, for instance due to spatial distribution or receptor clustering^25^. Similar to hNeo-4, mNeo-4 recapitulated the functionality of mIL-4 and induced STAT6 phosphorylation in A20 mouse B cells, albeit with 1,000-fold weaker potency (mIL-4 EC_50_ = 0.15 nM; mNeo-4 EC_50_ = 150 nM; Fig. 2b). Furthermore, hNeo-4 activated STAT6 phosphorylation on human monocyte-derived macrophages (MDMs; Extended Data Fig. 4a and Supplementary Table 6), and mNeo-4 activated primary mouse bone marrow-derived macrophages (BMDMs; Extended Data Fig. 4b and Supplementary Table 6). Interestingly, saturating concentrations of mNeo-4, but not hNeo-4, achieved full STAT6 phosphorylation activity on macrophages. Importantly, the parental design scaffold, Neo-2, did not induce STAT6 phosphorylation on IL-4-responsive cell lines (Extended Data Fig. 4a,b), and Neo-4, unlike Neo-2, did not induce activation of IL-2-mediated STAT5 phosphorylation on YT-1 human natural killer cells (Extended Data Fig. 4c).

STAT6 is most prominently activated by IL-4 and IL-13; however, these cytokines also induce weak phosphorylation of STAT1, STAT3 and STAT5 (refs. 3,26,27). hNeo-4 and mNeo-4 activated STAT1, STAT3 and STAT5 in human monocytes and mouse BMDMs, respectively (Extended Data Fig. 4i,j), albeit with much weaker magnitude than STAT6 activation. As for STAT6 activation, hNeo-4 activated STAT1, STAT3 and STAT5 less potently than both hIL-4 and hIL-13 on primary human monocytes, and mNeo-4 activated phosphorylated STAT1 (pSTAT1), pSTAT3 and pSTAT5 less potently than mIL-4 on primary mouse BMDMs. In contrast to its similar activation amplitude for STAT3 and STAT6, hNeo-4 induced STAT1 and STAT5 activation with a much weaker amplitude than natural cytokines. mNeo-4 induced activation of STAT6 and all three minor STATs to a similar extent as mIL-4.

Stimulation of primary human monocytes with either hIL-4 or hNeo-4 led to similar upregulation of M2-like macrophage signature genes, such as *CD209* and *CD200R1* (Fig. 2c and Extended Data Fig. 4d). Similarly, mNeo-4 mimicked the regulatory effects of mIL-4 on primary mouse BMDMs (Fig. 2d and Extended Data Fig. 4e), by both upregulating M2-like macrophage-associated genes, such as *Arg1* and *Chil3*, and downregulating M1-like macrophage-associated genes, such as *Il1b*. mNeo-4 also recapitulated the effects of mIL-4 on primary T cells from 4GET mice^28^, inducing IL-4 production indicative of T_H_2 polarization (Fig. 2e,f).

**Fig. 3 |:**
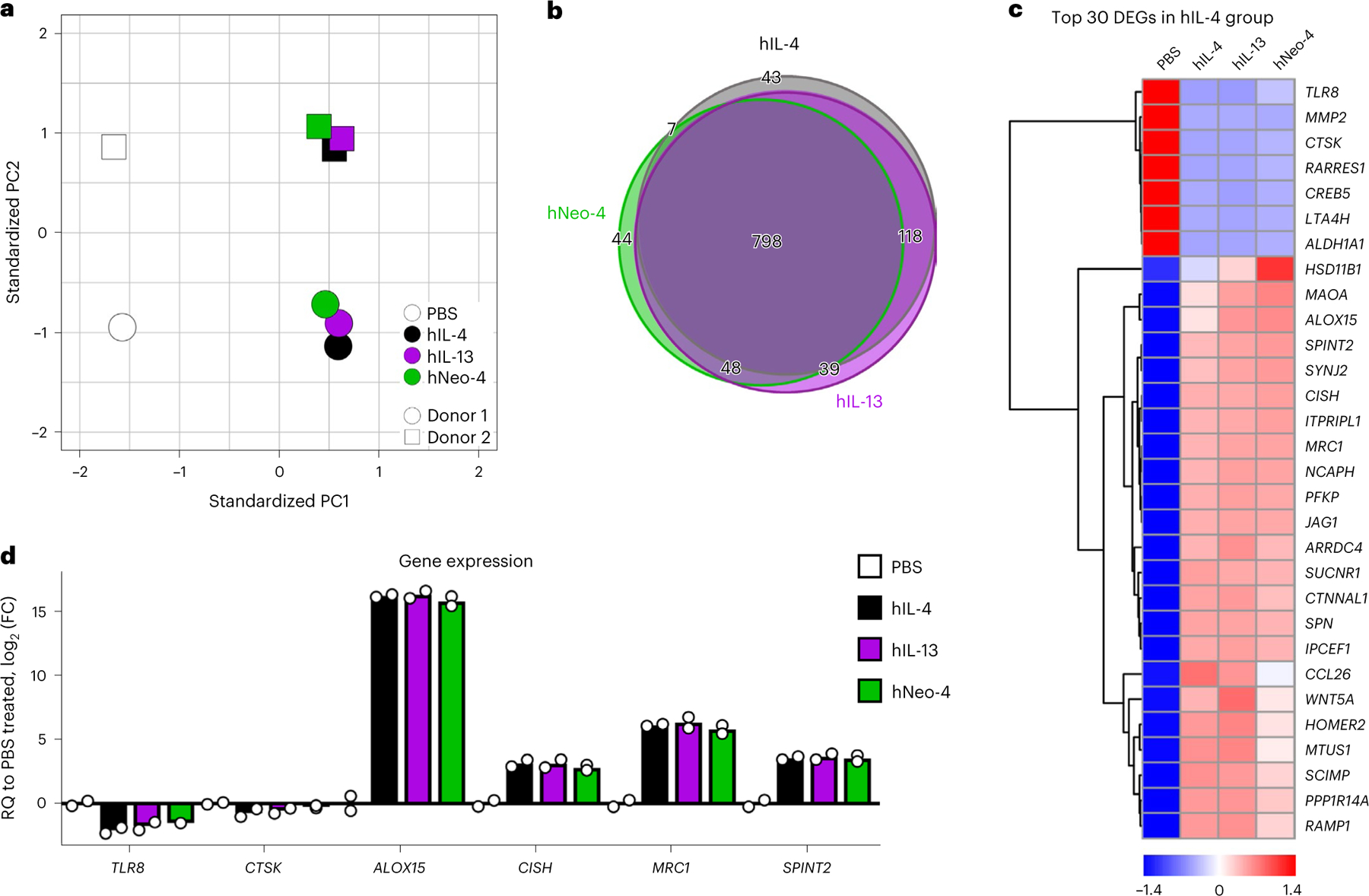
Genomic effects of hNeo-4 overlap with those of hIL-4 and hIL-13 in primary human monocytes. **a**, PCA of the gene expression profiles of primary human CD14^+^ monocytes after 24 h of treatment with hIL-4, hIL-13 or hNeo-4, as measured via RNA-seq (*n* = 2). **b**, Scaled Venn diagram depicting the number of differentially regulated genes in monocytes treated with hIL-4, hIL-13 or hNeo-4 in comparison to PBS-treated monocytes. **c**, Heat map showing the induction levels of the top 30 differentially expressed genes (DEGs) in IL-4-treated monocytes. **d**, RT–qPCR analysis of representative differentially regulated genes from RNA-seq analysis. Monocytes from two new donors were used.

To broadly probe the phenotypic effects of hNeo-4 compared to both hIL-4 and hIL-13, we conducted RNA sequencing (RNA-seq) on isolated primary human monocytes treated for 24 h with saturating cytokine concentrations (Fig. 3 and Extended Data Fig. 4f). Principal-component analysis (PCA) indicated that the gene induction profiles of monocytes treated with the three proteins differed substantially from the PBS control group but were similar to one another (Fig. 3a). Over 70% of differentially expressed genes compared to the control group (798 of 1,097 genes) were shared among hIL-4-, hIL-13- and hNeo-4-treated monocytes (Fig. 3b). In particular, hNeo-4 largely recapitulated the function of hIL-4, with 83% of the differentially regulated genes (805 of 966) shared between the cohorts, and the top 30 differentially expressed genes identified in the hIL-4 cohort were regulated to similar extents in the same directions by hNeo-4 and hIL-13 (Fig. 3c). Results were validated by quantitative reverse transcription PCR (qRT-PCR) analysis of representative genes in monocytes purified from new donors (Fig. 3d), reiterating the extensive overlap in gene programs elicited by hIL-4, hIL-13 and hNeo-4 in primary monocytes.

**Extended Data Fig. 5 |:**
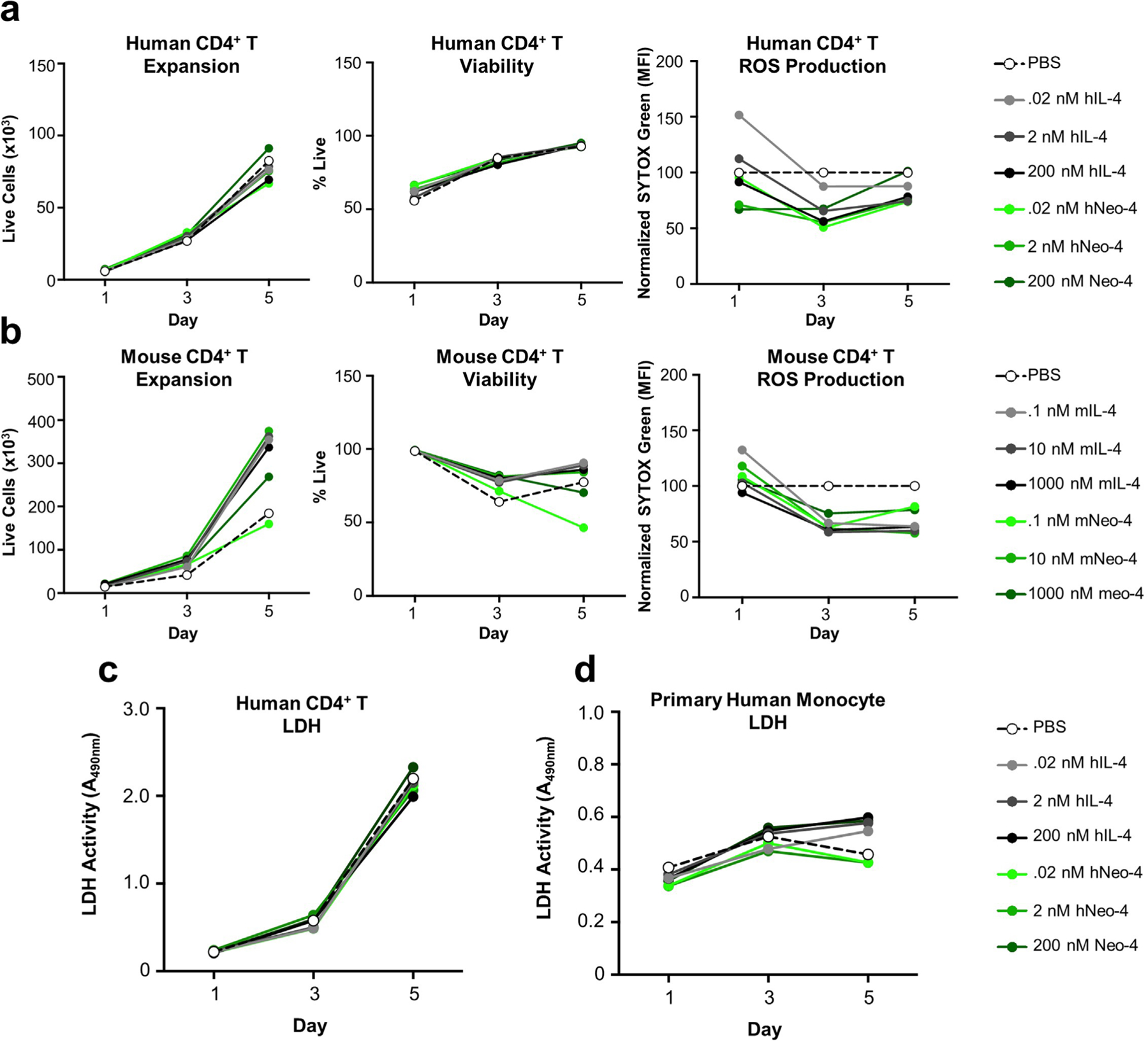
In vitro toxicity assessment of Neo-4 compared to IL-4. **a-b**, Human (**a**) or mouse (**b**) CD4^+^ T cells were subjected to Th2 polarization conditions through treatment with titrated amounts of IL-4 or Neo-4 for 1, 3, and 5 days. Live cell count, viability, and normalized reactive oxygen species staining are shown. Data represent mean ± SD (*n* = 2). **c-d**, LDH activity from supernatants of human CD4^+^ T cells (**c**) and human monocytes (**d**) polarized with titrated amounts of IL-4 or Neo-4 for 1, 3, and 5 days, as quantified by colorimetric assay. Data represent mean ± SD (*n* = 2).

To ensure that the sequence composition of Neo-4 did not lead to aberrant increases in toxicity, we analyzed T cell viability and reactive oxygen species production during T_H_2 polarization in response to natural versus engineered cytokines. Toxic molecules have detrimental effects on cell viability and induce aberrant increases in reactive oxygen species production^29^. We observed that Neo-4, like IL-4, did not negatively impact expansion or viability of polarized T cells (Extended Data Fig. 5a,b), even at saturating doses (Extended Data Fig. 4h). Furthermore, neither IL-4 nor Neo-4 led to noticeable increases in reactive oxygen species production. Additionally, high doses of hNeo-4 did not induce changes in lactate dehydrogenase (LDH) activity^30^ relative to IL-4 in either polarized T cells or monocytes (Extended Data Fig. 5c,d). Taken together, these results indicate that de novo engineered cytokines exhibit similar toxicity profiles as their natural counterparts.

### mNeo-4 upregulates the expression of M2-like macrophage-linked genes in vivo

**Extended Data Fig. 6 |:**
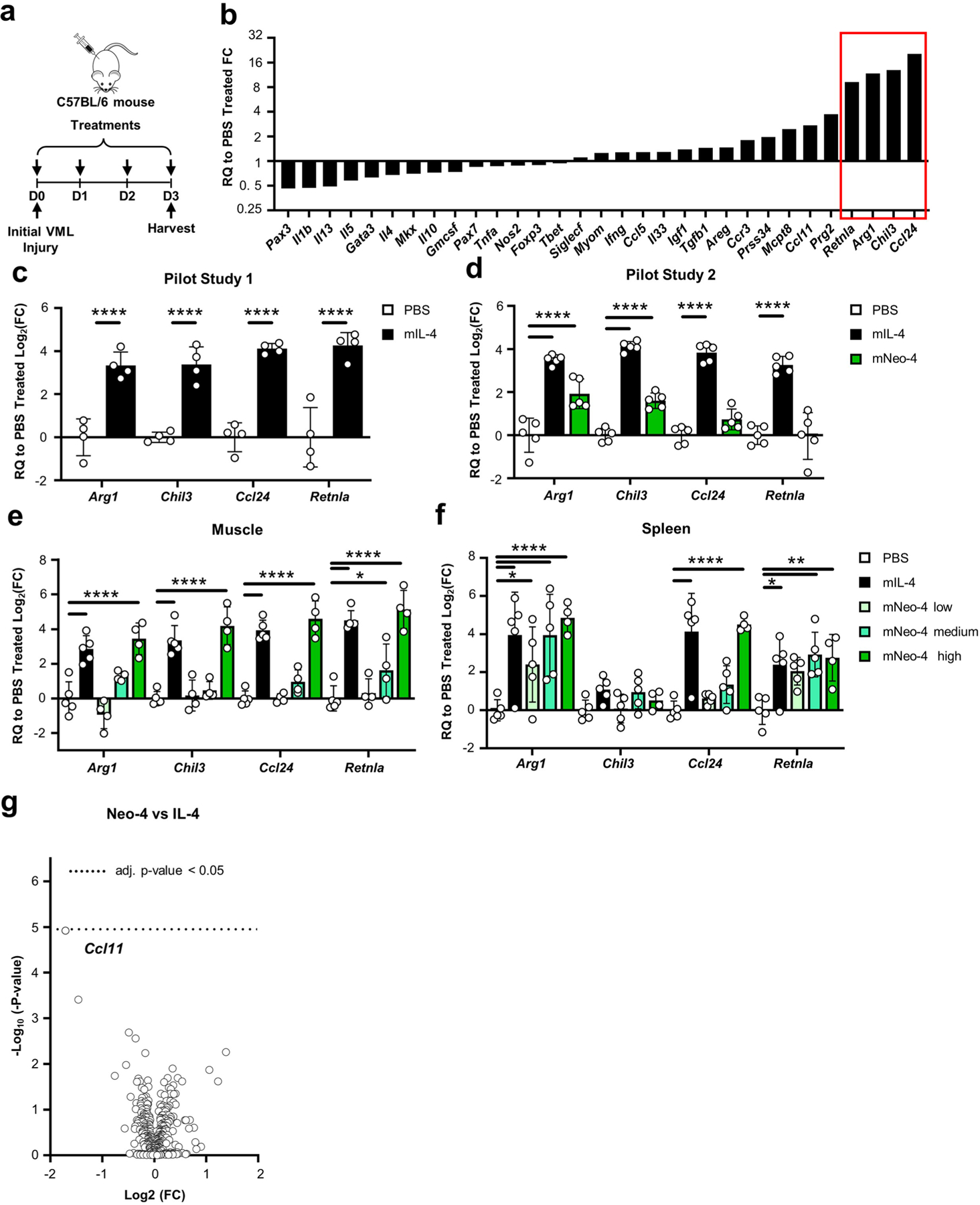
Functional characterization of mIL-4 and mNeo-4 in mouse volumetric muscle loss model. **a**, Illustration of experimental design and dosing regimen. Mice were treated with proteins or PBS for 4 consecutive days. Treatments were injected directly at the injury site on day 0, and the subsequent 3 treatments were injected subcutaneously. **b**, qRT-PCR analysis of genes related to tissue regeneration in isolated muscle samples (*n* = 1) from Pilot Study 1 (1.5 μg of mIL-4 or PBS treatments). Effects of mIL-4 were normalized to the PBS control. Fold change (FC) represents 2^−ΔΔCT^. **c**, Repeated qRT-PCR analysis of M2-like macrophage-related genes in isolated muscle samples (*n* = 4) from Pilot study 1. **d**, qRT-PCR analysis of M2-like macrophage-related genes from the isolated muscle samples (*n* = 5) in Pilot Study 2 (1.5 μg of mIL-4, 1.5 μg mNeo-4, or PBS treatments). Effects of mIL-4 or mNeo-4 were normalized to the PBS control. **e-f**, Mice were treated with either mNeo-4 (at low [0.03 μg, *n* = 5], medium [1.6 μg, *n* = 5], or high [71 μg, *n* = 4] dose), 1.5 μg mIL-4 (*n* = 5), or PBS (*n* = 5) in the model shown in **a**. qRT-PCR analysis of M2-like macrophage-related genes in isolated (**e**) muscle and (**f**) spleen samples are shown. Effects of each treatment were normalized to the PBS control. Data represent mean ± SD. *p < 0.05; ^**^p < 0.01; ^***^p < 0.001; ^****^p < 0.0001, two-way ANOVA. For ease of visualization, only significance compared to the control cohort is shown in panels **c-f**. All p-values are recorded in Supplementary Table 7. **g**, NanoString analysis (myeloid panel) on the muscle samples from mice treated as described in **e-f**. Volcano plots of differentially expressed genes are shown for the mNeo-4 versus the mIL-4 cohort. The significance of differential gene regulation was determined by calculating adjusted p values using the Benjamini-Yekutieli method of estimating false discovery rates (FDR) in the nCounter software (thresholds of adjusted P < 0.05, and 0.01 are shown).

**Fig. 4 |:**
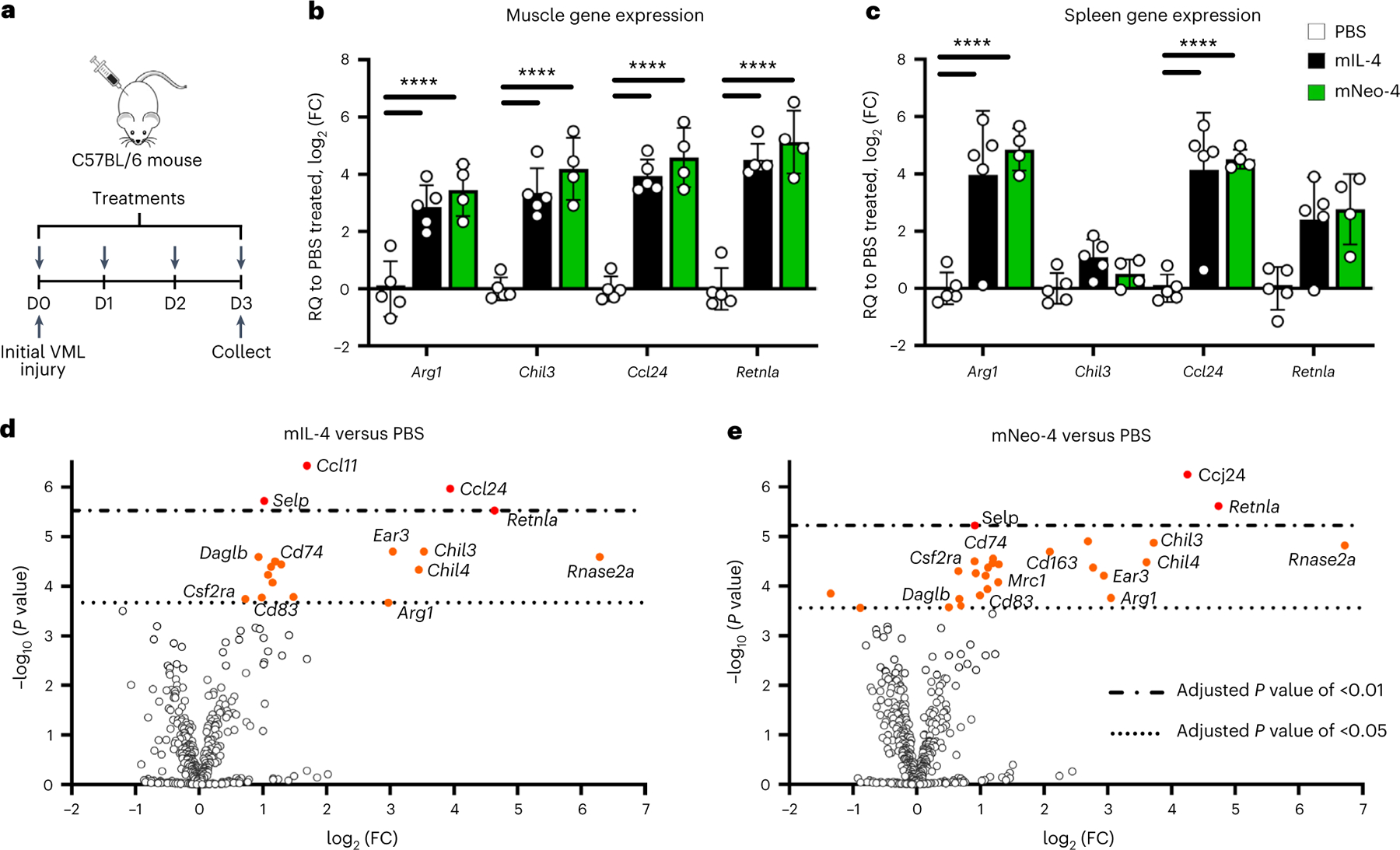
mNeo-4 recapitulates local and systemic restorative effects of mIL-4. **a**, Illustration of experimental design and dosing regimen for the mouse VML model. Mice were treated with mIL-4 (1.5 μg, *n* = 5), mNeo-4 (71 μg, *n* = 4) or PBS (*n* = 5) for 4 d consecutively. Treatments were injected directly at the injury site on day 0, and the subsequent three treatments were injected subcutaneously. **b**,**c**, RT–qPCR analysis of M2-like macrophage-related genes in the muscle (**b**) and spleens (**c**) of treated mice. FC represents 2^−ΔΔ*C*^_***t***_. Data represent mean ± s.d.; ****P < 0.0001. Data were analyzed by two-way ANOVA. **d**,**e**, NanoString analysis (myeloid panel) on the muscle samples from mice treated as described in **a**. Volcano plots of differentially expressed genes are shown for the mIL-4 versus PBS cohorts (**d**) and the mNeo-4 versus PBS cohorts (**e**). For ease of visualization, only significance compared to the control cohort is shown in **b** and **c**. The significance of differential gene regulation was determined by calculating adjusted *P* values using the Benjamini–Yekutieli method of estimating false discovery rates in the nCounter software (thresholds of adjusted *P* < 0.05 and *P* < 0.01 are shown). All *P* values are provided in Supplementary Table 7.

IL-4 stimulates proregenerative effects in the context of wound healing, particularly through promotion of M2-like macrophage polarization^31,32^. To characterize the in vivo function of computationally designed IL-4 mimetics, we studied the ability of mNeo-4 to reproduce the proregenerative effects of IL-4 in a mouse bilateral volumetric muscle loss (VML) model^33^. We first validated the effects of mIL-4 in a pilot study in which VML was induced on day 0, and mice were administered four daily doses of mNeo-4 followed by gene expression analysis in the quadricep muscle (Extended Data Fig. 6a). Among 31 regeneration-related genes that were screened, mIL-4 upregulated the expression of M2-like macrophage-related genes, including *Arg1*, *Chil3*, *Ccl24* and *Retnla* (Extended Data Fig. 6b,c and Supplementary Table 7). We repeated this experiment comparing equal doses of mIL-4 and mNeo-4 (Extended Data Fig. 6d). mNeo-4 mimicked the upregulation of M2-like macrophage-related genes induced by mIL-4 in the VML model, although activity was inferior due to its weaker potency. Dose optimization revealed that 71 μg of mNeo-4 per mouse per day induced similar proregenerative gene levels in the muscle as did 1.5 μg of mIL-4 per mouse per d (Fig. 4a,b, Extended Data Fig. 6e and Supplementary Table 7). mNeo-4 also mimicked the systemic effects of mIL-4, upregulating the expression of representative M2-like macrophage-associated genes in the spleen (Fig. 4c, Extended Data Fig. 6f and Supplementary Table 7). NanoString analysis was conducted on muscle samples from mice treated with PBS, 1.5 μg of mIL-4 per mouse per day or 71 μg of mNeo-4 per mouse per day. Among the 624 myeloid panel genes screened, the profiles of mNeo-4 and mIL-4 were largely overlapping (Fig. 4d,e), and no genes reached significance for differential regulation (Extended Data Fig. 6g). The top regulated genes were also consistent with RT–qPCR analyses. Collectively, these studies highlighted the functional similarity between mIL-4 and mNeo-4 in the context of muscle injury.

### hNeo-4 mediates biased signaling through type I receptors

**Extended Data Fig. 7 |:**
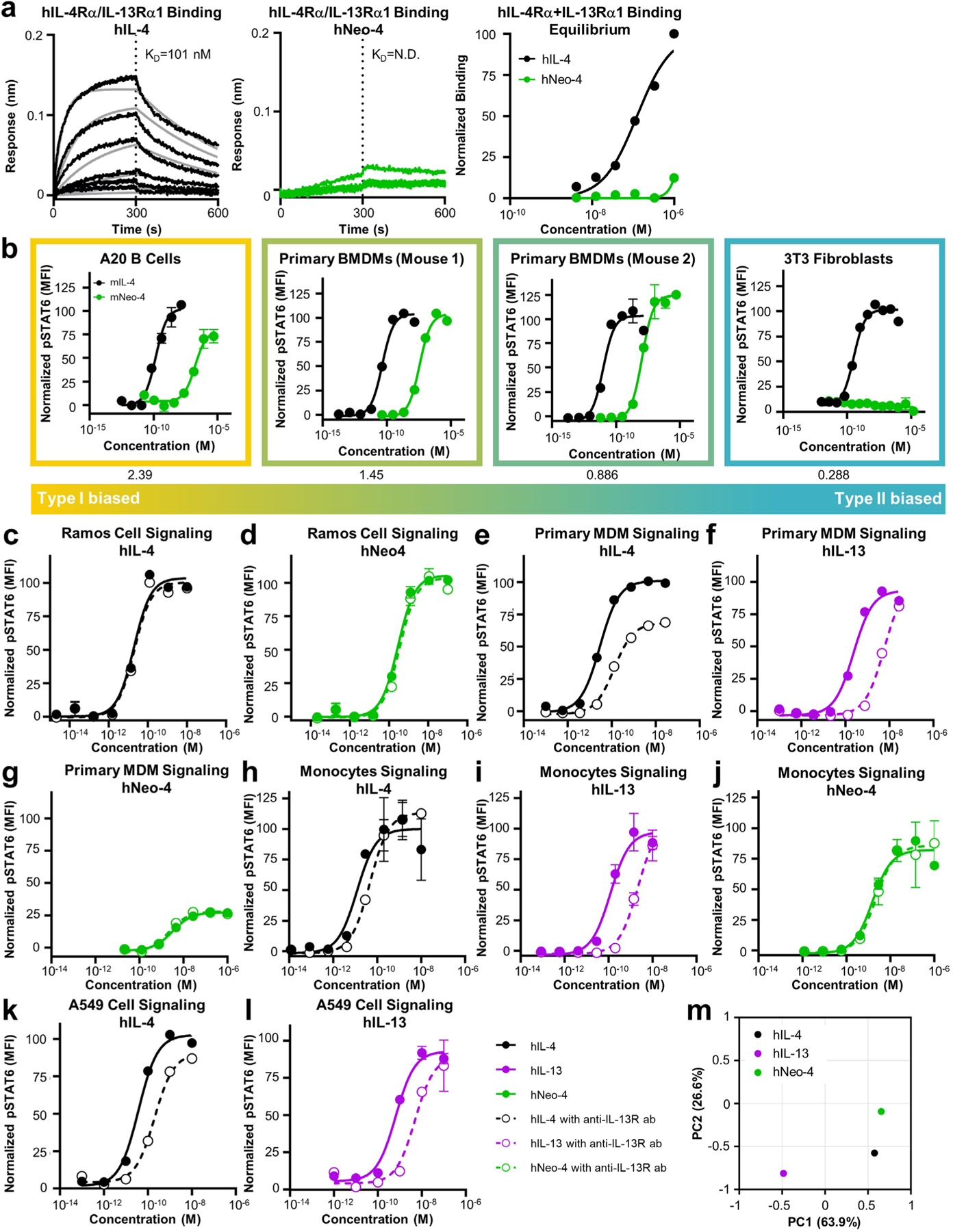
Evaluation of biased signaling activation induced by IL-4 mimetics. **a**, BLI sensograms depicting interactions between hIL-4 or hNeo-4 (3-fold serial dilutions starting at 1 μM) and the hIL-4Rα/hIL-13Rα1 complex. Equilibrium BLI titrations of hIL-4 or hNeo-4 against the hIL-4Rα/hIL-13Rα1 complex are shown at *right*. Raw data were fitted using a 1:1 Langmuir binding model. Fitted curves are shown in gray. **b**, STAT6 activation elicited by mIL-4 or mNeo-4 in mouse A20 cells (*n* = 3), BMDMs Mouse 1 (*n* = 3), BMDMs Mouse 2 (*n* = 2), and 3T3 fibroblast cells (*n* = 3). Type I or Type II receptor biases were defined by the ratio of mγ_c_ to mIL-13Rα1 expression on each cell line (shown below each plot), with higher ratios indicating type I receptor bias. Data represent mean ± SD. **c-d**, STAT6 activation induced by (**c**) hIL-4 and (**d**) hNeo-4 in human Ramos B cells (*n* = 3) in the presence (dotted lines) or absence (solid lines) of anti-IL-13Rα1 antibody. **e-g**, STAT6 activation induced by (**e**) hIL-4, (**f**) hIL-13, or (**g**) hNeo-4 in primary human monocyte-derived macrophages (MDMs) (*n* = 2) in the presence (dotted lines) or absence (solid lines) of anti-IL-13Rα1 antibody. **h-j**, STAT6 activation induced by (**h**) hIL-4, (**i**) hIL-13, or (**j**) hNeo-4 in primary human monocytes (*n* = 3) in the presence (dotted lines) or absence (solid lines) of anti-IL-13Rα1 antibody. **k-l**, STAT6 activation induced by (**k**) hIL-4 or (**l**) hIL-13 in A549 lung epithelial cells (*n* = 3) in the presence (dotted lines) or absence (solid lines) of anti-IL-13Rα1 antibody. Data represent mean ± SD. **m**, Principal component analysis (PCA) of STAT6 activation induced by hIL-4, hIL-13, and hNeo-4 on 5 human cell lines (Ramos B cells, primary monocytes, MDMs, primary fibroblasts, and A549 cells). Half-maximal effective concentrations (EC_50_) that were used to compare the signaling activities of the 3 cytokines are tabulated in Supplementary Table 6.

**Fig. 5 |:**
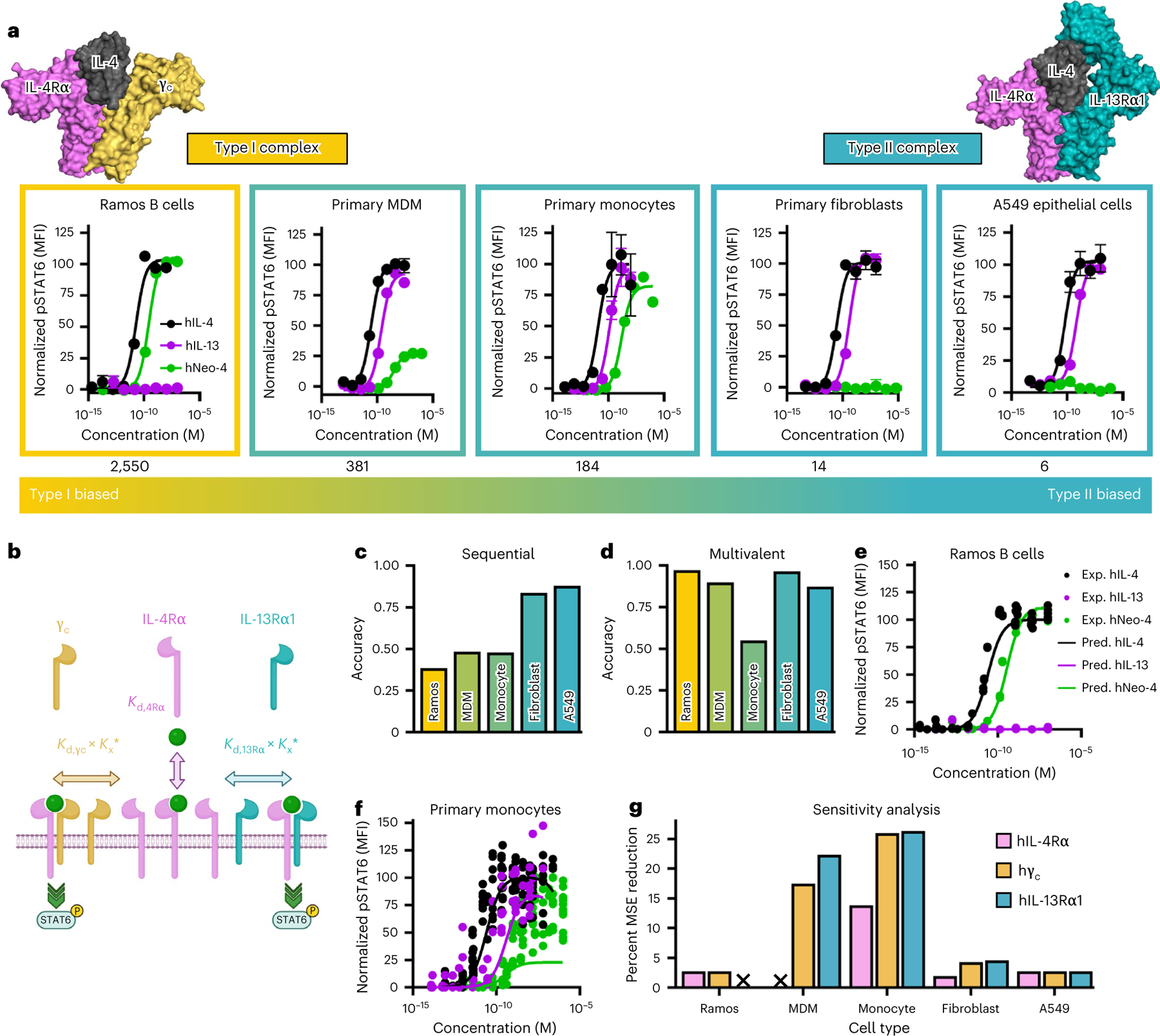
hNeo-4 biases toward type I receptor activation and is a valuable tool for elucidating IL-4 signaling mechanisms. **a**, STAT6 activation elicited by hIL-4, hIL-13 or hNeo-4 in human Ramos B cells (*n* = 3), primary monocytes (*n* = 3), MDMs (*n* = 2), primary fibroblasts (*n* = 3) and A549 lung epithelial cells (*n* = 3). Type I or type II receptor biases were defined by the ratio of hγ_c_ to hIL-13Rα1 expression on each cell line (shown below each plot), with higher ratios indicating type I receptor bias. **b**, Illustration of the multivalent IL-4 signaling model. Data represent mean ± s.d.; *K*_x_*, crosslinking constant. **c**,**d**, Prediction accuracies for the five cell types shown in **a**, using either the sequential (**c**) or multivalent IL-4 (**d**) signaling model. **e**,**f**, Experimental (Exp.) STAT6 activation by hIL-4, hIL-13 or hNeo-4 compared to multivalent model predictions (Pred.) in Ramos B cells (**e**) or primary monocytes (**f**). **g**, Sensitivity test evaluating the impact of hIL-4Rα, hγ_c_ or hIL-13Rα1 expression fluctuations on the prediction accuracy of multivalent model. MSE, mean squared error. The ‘×’ symbol indicates receptors for which surface expression was not detectable by flow cytometry, and thus analyses were not included in sensitivity tests.

Native IL-4 signals through both type I and type II complexes, which share the IL-4Rα chain. Thus, IL-4 activity varies depending on the cell surface expression of γ_c_ (type I complex) versus IL-13Rα1 (type II complex^3^; Fig. 5a, Extended Data Fig. 7b and Supplementary Tables 8 and 9). Neo-4 cytokines were derived from the Neo-2 scaffold, which engages the IL-2Rβ–γ_c_ heterodimer, and hNeo-4 and mNeo-4 were designed and affinity matured to engage γ_c_, without consideration for IL-13Rα1 binding. Consequently, we anticipated that hNeo-4 would exclusively engage the type I complex. BLI binding studies confirmed that hNeo-4 did not interact with the type II complex (Extended Data Fig. 7a). Signaling assays were performed on cell lines displaying a range of type I versus type II receptors, including Ramos B cells, primary human monocytes, primary human MDMs, primary human fibroblasts and A549 epithelial cells, to compare the activation profiles of hNeo-4 to those of the natural hIL-4 and hIL-13 cytokines (Fig. 5a and Supplementary Table 8). Because we lack information on the partitioning of IL-4Rα between type I and type II complexes, we report type I versus type II receptor biases based on the hγ_c_:hIL-13Rα1 expression ratio of each cell line, although either co-receptor partner could be limiting. As expected, hIL-4 potently induced STAT6 phosphorylation in all cell lines (Fig. 5a), whereas hIL-13 only activated cell types with considerable type II complex proportions (Fig. 5a and Supplementary Table 8). hNeo-4 mimicked hIL-4 activity on cells that predominantly express the type I complex, but hNeo-4 exhibited inferior potency and amplitude on cell types with lower type I complex proportions and was inert on cells that predominantly express the type II complex. Similar type I complex-biased behavior was observed for mNeo-4 signaling (Extended Data Fig. 7b and Supplementary Table 9). To confirm that Neo-4 exclusively engages the type I complex, we conducted signaling studies in the presence of a neutralizing antibody to hIL-13Rα1 (Extended Data Fig. 7c–l). As expected, anti-hIL-13Rα1 did not interfere with hIL-4 and hNeo-4 on Ramos cells, which primarily express the type I complex (Extended Data Fig. 7c,d), but the antibody attenuated signaling of hIL-4 and hIL-13, but not hNeo-4, on primary MDMs, primary monocytes and A549 epithelial cells (Extended Data Fig. 7e–l). These findings reveal that Neo-4 molecules exhibit distinct signaling biases compared to natural cytokines by exclusively engaging the type I complex.

PCA of STAT6 signaling data from each of the five treated cell lines (Extended Data Fig. 7m) revealed that, as anticipated, hIL-13 (type II complex only) and hNeo-4 (type I complex only) were the most different from one another, as reflected by their distance along PC1. hIL-4 was intermediate between hIL-13 and hNeo-4, with a PC1 value closer to that of hNeo-4, indicating that the IL-4 signaling profile agreed more closely with the type I receptor-biased hNeo-4.

**Extended Data Fig. 8 |:**
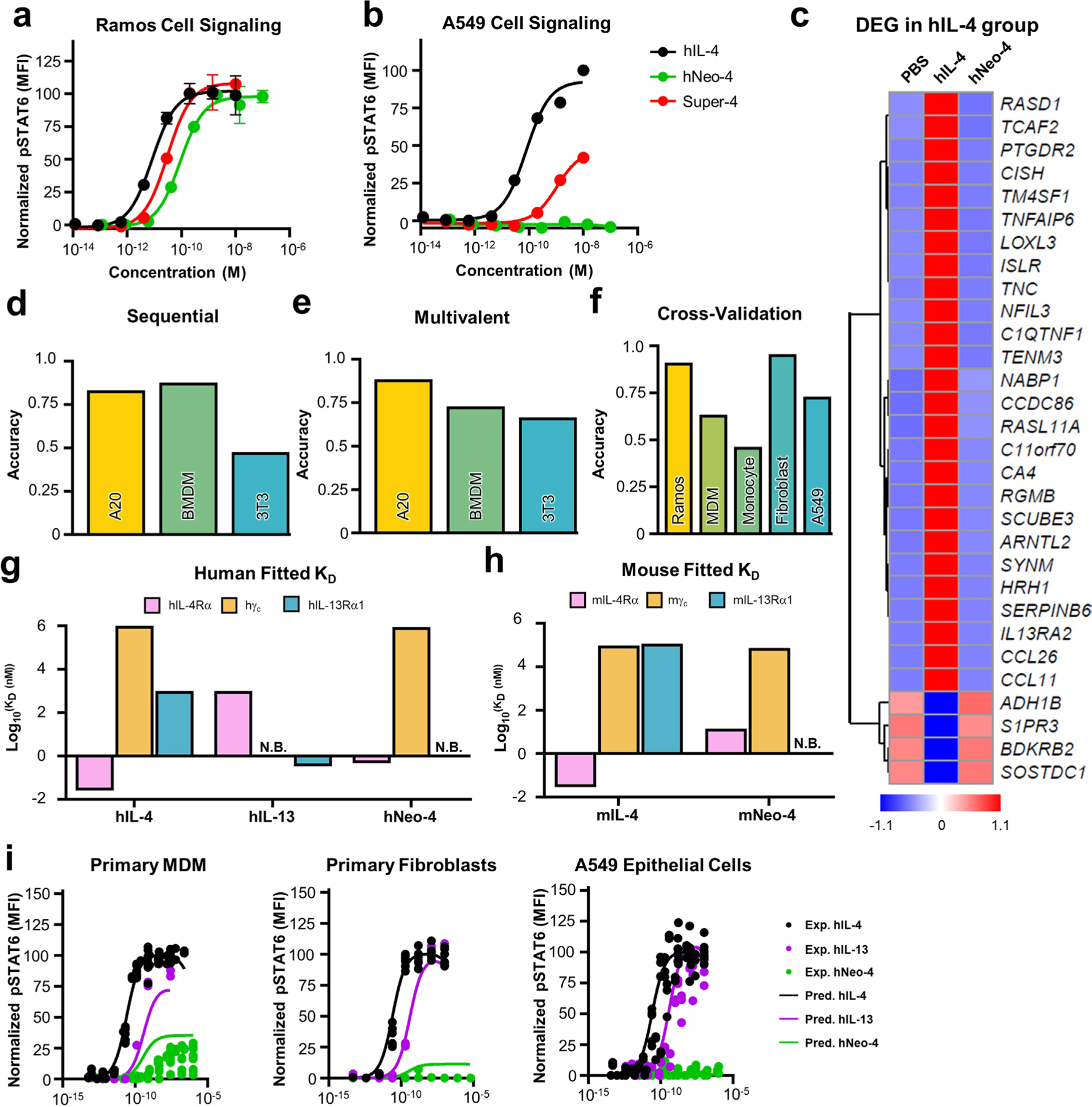
Characterization and modeling of biased type I IL-4 receptor signaling elicited by hNeo-4. **a-b**, STAT6 phosphorylation induced by hIL-4, hNeo-4, and super-4 in (**a**) Ramos B cells and (**b**) A549 lung epithelial cells. Data represent mean ± SD (*n* = 3 biologically independent samples). **c**, Heat map showing the induction levels of the top 30 differentially expressed genes (DEGs) in IL-4-treated fibroblasts. **d-e**, Prediction accuracies for STAT6 activation induced by mNeo-4 or mIL-4 in A20 B cells, primary bone marrow-derived macrophages (BMDMs), and 3T3 fibroblast cells, using either the (**d**) sequential or (**e**) multivalent IL-4 signaling model. **f**, Cross-validation of the accuracies of the multivalent model STAT6 activation predictions in human Ramos B cells, primary monocytes, primary monocyte-derived macrophages (MDMs), primary fibroblasts, and A549 lung epithelial cells. **g**, Fitted equilibrium dissociation constants (K_D_) of hIL-4, hIL-13, or hNeo-4 binding to IL-4 receptor chains obtained from the multivalent model. N.B. indicates no binding. **h**, Fitted K_D_ values of mIL-4 or mNeo-4 binding to IL-4 receptor chains obtained from the multivalent model. **i**, Experimental STAT6 activation by hIL-4, hIL-13, or hNeo4 compared to multivalent model predictions in MDMs, primary fibroblasts, and A549 cells.

Previously, an hIL-4 mutant (denoted super-4) was evolved through affinity maturation of the natural hIL-4 cytokine toward hγ_c_, which led to biased activation of type I complexes^5^. We compared the signaling behavior of super-4 to hNeo-4 on type I (Ramos) and type II (A549) complex-expressing cell lines (Extended Data Fig. 8a,b). Whereas both super-4 and hNeo-4 activated Ramos cells (Extended Data Fig. 8a), super-4 retained signaling activity (tenfold weaker than hIL-4) on A549 cells and hNeo-4 was completely inert (Extended Data Fig. 8b), illustrating that computational design, but not directed evolution, led to cleanly biased type I signaling. RNA-seq studies on human primary fibroblasts further corroborated the exclusive type I complex activity of hNeo-4 (Extended Data Fig. 8c). As anticipated, hIL-4 strongly upregulated inflammation-related genes;^31^ however, hNeo-4-treated cells behaved identically to the PBS control cohort (Extended Data Fig. 8c).

### Mechanistic model elucidates nuanced hNeo-4 signaling

Type I versus type II complex expression patterns were generally predictive of hNeo-4 cell signaling behavior; however, we observed unexpectedly low hNeo-4 activity on primary MDMs despite their 381:1 ratio of type I (γ_c_) to type II (IL-13Rα1) co-receptors (Fig. 5a). To comprehensively explore the relationship between receptor expression, ligand affinities and signaling, we used STAT6 activation and surface receptor quantification data to develop mechanistic computational models (Fig. 5b). Because spatiotemporal organization of cytokine receptors in the membrane is not fully understood, we attempted both a classic sequential binding model^34^ and a recently developed multivalent model^35^. The sequential binding model assumed that the initial binding events for IL-4/Neo-4 and IL-13 were with their private receptors (IL-4Rα and IL-13Rα1, respectively), followed by co-receptor recruitment. The multivalent model instead assumed that ligands could bind to their respective receptors in either order. We found that the more flexible multivalent model matched signaling responses of five human cell types (Fig. 5c,d) and three mouse cell types (Extended Data Fig. 8d,e) with greater accuracy. Cross-validation studies confirmed the predictive accuracy of the multivalent model (Extended Data Fig. 8f), and the fitted *K*_d_ values from the multivalent model (based on input signaling data) converged toward known trends^4^ and our BLI study data (Extended Data Fig. 8g,h and Supplementary Table 5). Overall, these results showed that the multivalent model outperformed the sequential binding model and was consistent with experimental results.

Although the multivalent model generally aligned with experimental signaling data, accuracy was lower for primary MDMs and monocytes (Fig. 5e,f and Extended Data Fig. 8i). To probe this discrepancy, we investigated the sensitivity of the multivalent model to expression levels of IL-4Rα, γ_c_ and IL-13Rα1 by refitting the model while varying receptor densities (Fig. 5g). Fitting accuracy was most sensitive to variation in hγ_c_ and IL-13Rα1 chain expression, consistent with previous reports using IL-4 variants^5^. The model was particularly sensitive to hγ_c_ and IL-13Rα1 abundance on primary MDMs and monocytes, suggesting that receptor expression alone may not be sufficient to predict myeloid cell signaling responses.

### Thermal stability of IL-4 mimetics enables 3D printing

**Extended Data Fig. 9 |:**
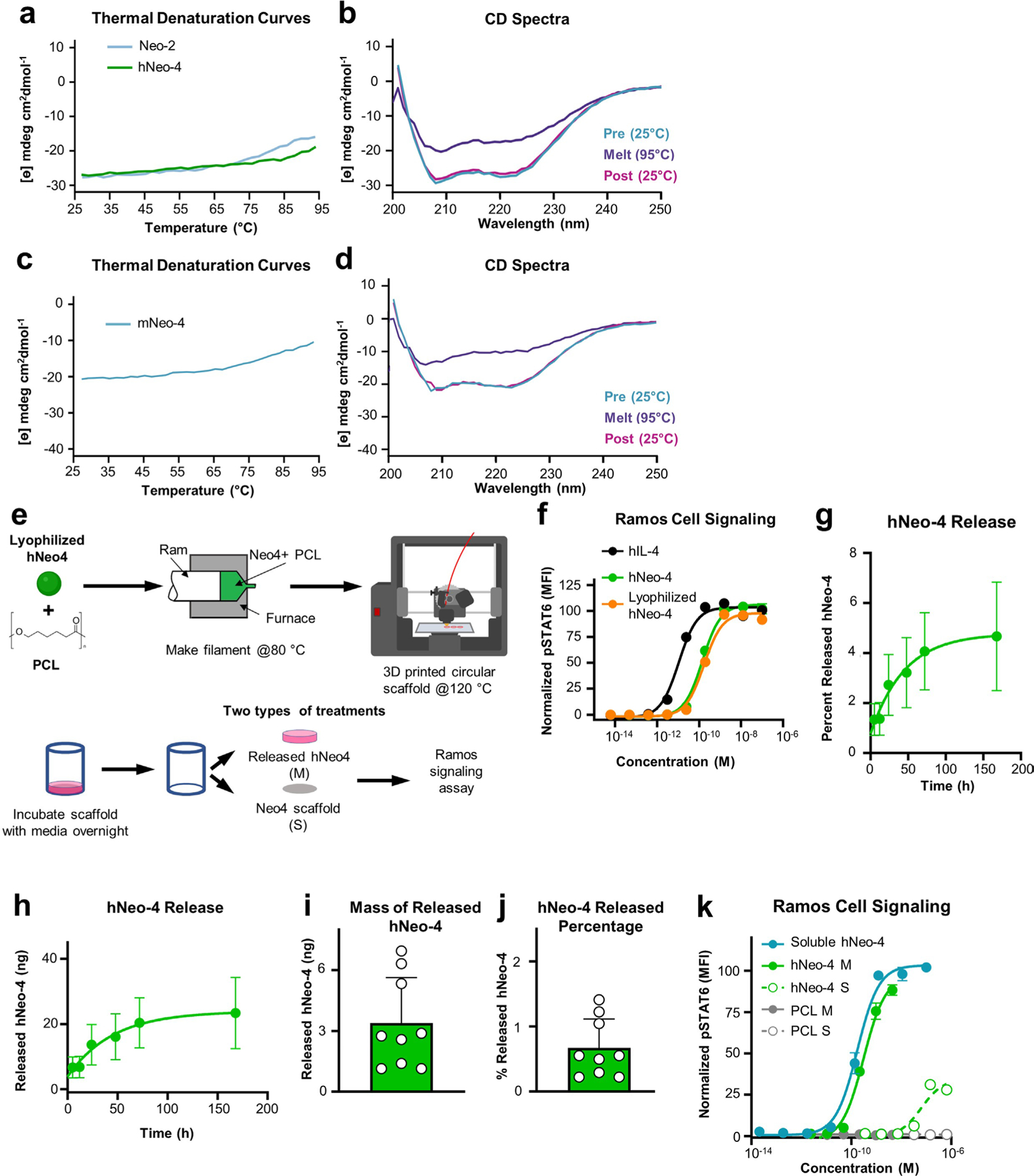
Thermal stability characterization of IL-4 mimetics. **a**, Thermal denaturation curves for hNeo-4 and Neo-2. **b**, Circular dichroism (CD) spectra of hNeo-4 at pre-heating (25 °C), melting (95 °C), and post-heating (25 °C) stages. **c**, Thermal denaturation curve for mNeo-4. **d**, CD spectra of mNeo-4 pre-heating (25 °C), melting (95 °C), and post-heating (25 °C) stages. **e**, Schematic of the manufacturing and characterization ofa PCL-based 3D-printed scaffold incorporating hNeo-4. **f**, STAT6 phosphorylation induced by hIL-4, hNeo-4, and lyophilized hNeo-4 in Ramos B cells. Data represent mean ± SD (*n* = 2) **g**, Release profile of hNeo-4 (percentage mass) from 3D-printed scaffolds, as measured by fluorescent protein quantification assay. Data represent mean ± SD (*n* = 3 biologically independent samples). **h**, Release profile of hNeo-4 (mass) from 3D-printed scaffolds, as measured by fluorescent protein quantification assay. Data represent mean ± SD (*n* = 3 biologically independent samples). **i**, Quantification of hNeo-4 mass released from 3D-printed scaffold over 16 h incubation at 4 °C, as measured by fluorescent protein quantification assay. Data represent mean ± SD (*n* = 9 biologically independent samples). **j**, Percentage mass of hNeo-4 released from 3D-printed scaffold over 16 h incubation at 4 °C, as calculated based on the measurements from fluorescent protein quantification assay shown in **i**. Data represent mean ± SD (*n* = 9). **k**, STAT6 signaling activation induced by hNeo-4 from 3D-printed scaffolds on Ramos B cells. Signaling stimulated by either hNeo-4 released from 3D-printed scaffolds (M) or hNeo-4 on the surface of 3D-printed scaffolds (S) is shown. Signaling of unmodified PCL scaffolds is shown for reference. Data represent mean ± SD (*n* = 3 biologically independent samples).

**Fig. 6 |:**
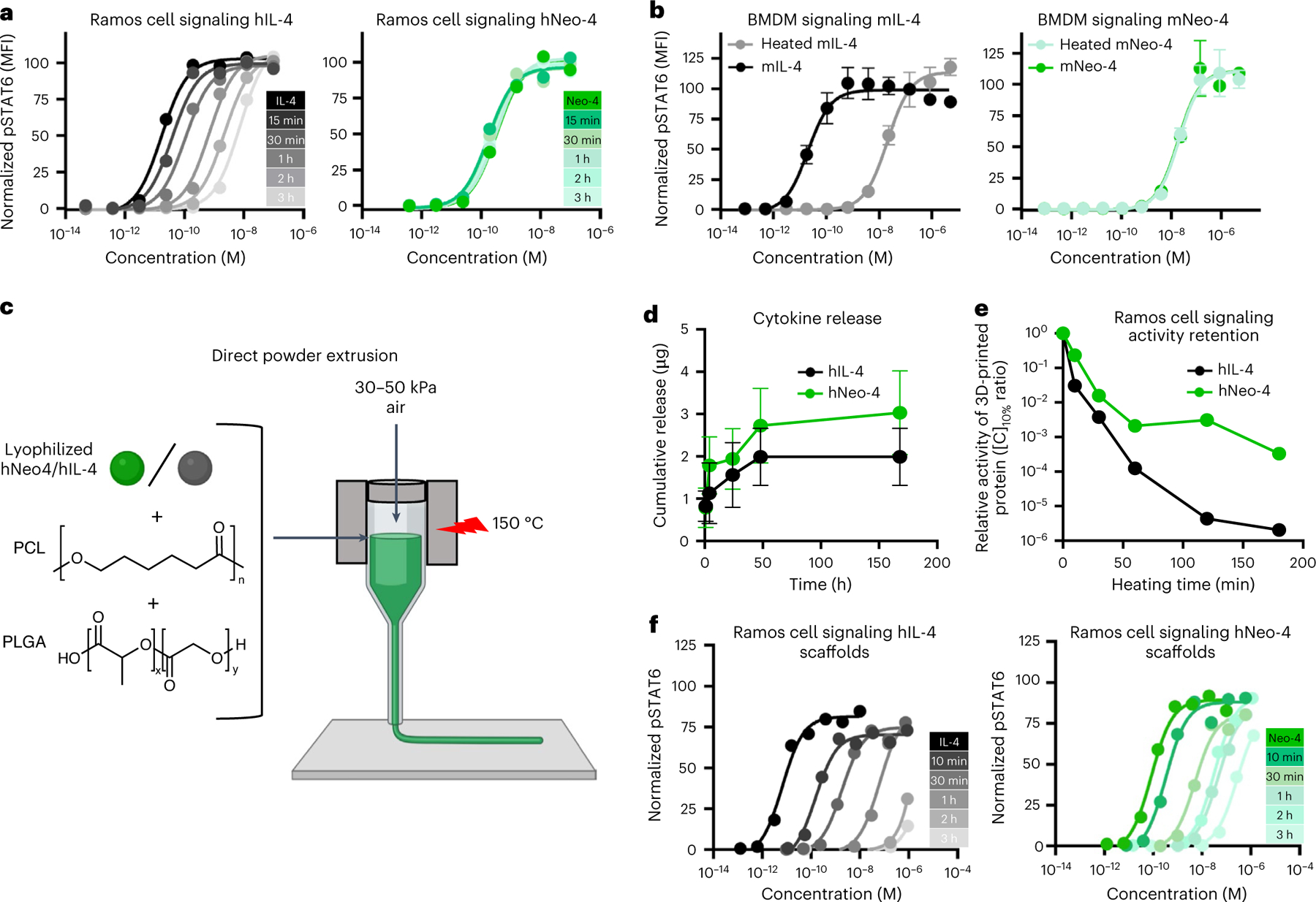
IL-4 mimetics are thermally stable and can be directly incorporated into 3D-printing processes. **a**, STAT6 activation induced by heated or unheated hIL-4 (left) and hNeo-4 (right) on Ramos cells. Heat treatments were performed at 95 °C for the indicated lengths of time before signaling assays. Data represent mean ± s.d. (*n* = 2 biologically independent samples). **b**, STAT6 activation elicited by heated or unheated mIL-4 (left) and mNeo-4 (right) on mouse BMDMs. Heat treatments were performed at 95 °C for 60 min before signaling assays (*n* = 3 biologically independent samples). **c**, Schematic depicting the manufacturing of 3D-printed scaffolds using direct powder extrusion. **d**, Release profile of hNeo-4 and hIL-4 from 3D-printed scaffolds, as measured by fluorescent protein quantification assay. Data represent mean ± s.d. (*n* = 4 biologically independent samples). **e**, Activity retention of released hIL-4 and hNeo-4 from scaffolds after the polymer was subjected to a prolonged heating process and printed. Activity is plotted as the ratio of the concentration required to achieve 10% maximal IL-4-induced STAT6 activation on Ramos cells ([C]_10%_) for untreated cytokine to that of the released cytokine from a 3D-printed scaffold heated for the indicated length of time. Each point indicates the composite curve fit from two pooled biologically independent samples. Full signaling titrations are shown in **f**. **f**, STAT6 signaling activation on Ramos cells induced by untreated cytokine and released cytokine from a 3D-printed scaffold heated for the indicated length of time for hIL-4 (left) and hNeo-4 (right). Heat treatments were performed in the melting chamber of the 3D printer at 150 °C for the indicated durations before printing and subsequent release over 48 h. Data represent mean ± s.d. (*n* = 2 biologically independent samples).

Neo-2, the parental scaffold of both hNeo-4 and mNeo-4, was designed to be thermostable and retained bioactivity after extreme heating^17^. Hyperstability of hNeo-4 was demonstrated by circular dichroism (CD) spectroscopy studies, as the molecule completely recovered ellipticity after heating to 95 °C followed by cooling to 25 °C (Extended Data Fig. 9a), and the thermal melting curves of hNeo-4 and Neo-2 showed direct overlap (Extended Data Fig. 9b). hNeo-4-derived mNeo-4 also showed robust stability by CD spectroscopy and thermal melting curve analysis (Extended Data Fig. 9c,d). We heated hIL-4 and hNeo-4 to 95 °C for various time periods and subjected the proteins to Ramos cell signaling assays (Fig. 6a and Supplementary Table 6). Unlike hIL-4, which progressively lost potency over time, hNeo-4 activity was unchanged after 3 h of heating. Similarly, mNeo-4 activity was robust to 1 h of heating at 95 °C, whereas mIL-4 lost 1,000-fold activity on mouse BMDMs (Fig. 6b).

We wondered whether the thermal stability of Neo-4 could be exploited to allow direct inclusion in 3D-printed biomaterial scaffolds. Three-dimensional printing is one of the most versatile tools for biomaterial fabrication due to the rapid and precise generation of complex designs for controlled biomolecule delivery^36^. Hot-melt extrusion (HME) is used to achieve homogenous molecular dispersion, precluding inclusion of heat-sensitive biomolecules into 3D-printed scaffolds^37,38^, as most polymers used for printing have melting temperatures exceeding 100 °C (ref. 39). We conjectured that the remarkably thermostable hNeo-4 could survive harsh heat processing during 3D printing. We fabricated filaments with either polycaprolactone (PCL) only or PCL plus lyophilized hNeo-4 through HME at 80 °C and printed them on a 3D printer at 120 °C (Extended Data Fig. 9e). hNeo-4 potency was confirmed to be unchanged by lyophilization (Extended Data Fig. 9f). hNeo-4 exhibited a burst release pattern from the scaffolds; however, only 5% of the protein was released over 7 d (Extended Data Fig. 9g,h), likely due to slow degradation of PCL in physiological environments^40^. Ramos cell stimulation studies performed on conditioned medium or the printed scaffolds showed that hNeo-4 induced STAT6 phosphorylation in both released and scaffold-embedded formats (Extended Data Fig. 9k). Released hNeo-4 showed strikingly similar potency to soluble hNeo-4, demonstrating that the functional activities of hNeo-4 were highly preserved following intense thermal treatments.

**Extended Data Fig. 10 |:**
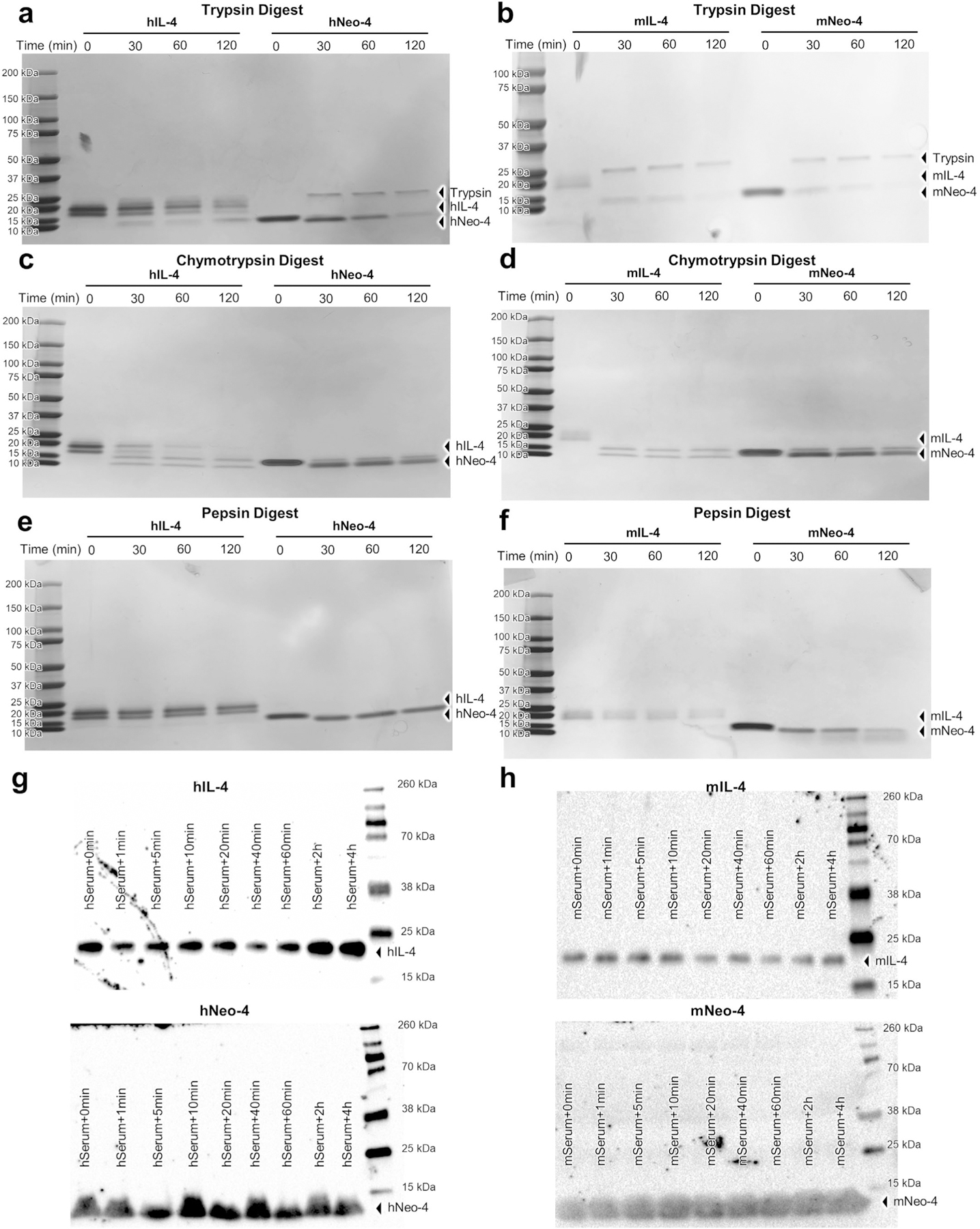
Characterization of IL-4 and Neo-4 protease and serum stability. **a-f**, Human or mouse IL-4 and Neo-4 were incubated with trypsin (**a,b**), chymotrypsin (**c,d**), or pepsin (**e,f**) for various time periods and subsequently subjected to SDS-PAGE analysis to detect protein degradation. Protease stability tests of each protein were performed once for each time point. **g**, 1 mg/ml (final concentration) of 6XHis-tagged hIL-4 or hNeo-4 was incubated in human serum for the indicated time periods up to 4 h. **h**, 1 mg/ml (final concentration) of 6XHis-tagged mIL-4 or mNeo-4 was incubated in mouse serum for the indicated time periods up to 4 h. Cytokines were detected with an anti-6XHis tag antibody via immunoblotting. hIL-4 and mIL-4 migrate as 22–24 kDa and 19–23 kDa, respectively, on the gel due to glycosylation. Serum stability test of each protein was performed once independently with similar results.

To improve cytokine release and highlight the advantages for our hyperstable IL-4 mimetic, we used direct powder extrusion 3D printing to produce PCL- and poly(d,l-lactide-co-glycolide)-blended (PCL:PLGA) scaffolds containing hIL-4 versus hNeo-4. PCL and PLGA are widely used biocompatible thermoplastic polymers, with PCL bolstering mechanical integrity and PLGA degrading faster to enhance material release^41,42^. Scaffolds were printed at 150 °C (Fig. 6c) and subjected to prolonged heating in the melting chamber to illustrate how the cytokine potency might vary for longer prints, which is required for preparing larger scaffolds. PCL:PLGA blend scaffolds exhibited greater protein release than PCL scaffolds (Fig. 6d). STAT6 activation studies on Ramos cells revealed that, remarkably, released hNeo-4 fully activated signaling with a 1,000-fold loss in potency after 3 h of heating, whereas released hIL-4 did not fully activate STAT6 signaling even at micromolar concentrations and exhibited a 1,000,000-fold loss in potency following equivalent treatment (Fig. 6e). Furthermore, hNeo-4 was more robust to potency loss than hIL-4 throughout the heating time course (Fig. 6f). These studies showcased how the enhanced thermal stability of hNeo-4 compared to hIL-4 empowers the direct incorporation of cytokines into 3D-printing processes. It is important to note that although hNeo-4 and mNeo-4 were designed for thermostability, the molecule was not engineered for superior protease or serum stability, and no changes in these properties were observed compared to the respective natural cytokines (Extended Data Fig. 10).

## Discussion

In contrast to naturally derived proteins, the exquisite thermodynamic stability of de novo computationally designed proteins makes them robust to amino acid mutations on their surface^17,18^, allowing facile generation of modified de novo proteins with alternative specificities and tunable affinities using existing scaffolds. We designed IL-4 protein mimetics from Neo-2 by introducing 16 amino acid mutations, remodeling its interface with IL-2Rβ to specifically and tightly bind IL-4Rα with no residual IL-2 receptor engagement. Unlike natural cytokines, which may lose structural stability after sequence modification^19^, Neo-2 maintained its stability when engineered into hNeo-4 and mNeo-4. hNeo-4 also had high solubility, allowing concentration to >50 μM in PBS without aggregation after several freeze–thaw cycles. Furthermore, hNeo-4 and mNeo-4 faithfully recapitulated binding, STAT6 (and other minor STAT) phosphorylation, gene regulation, cell differentiation and systemic immune effects induced by the respective natural cytokines, demonstrating the potential to design mimetics for an unlimited range of systems using existing platforms. Preliminary toxicity studies indicated that Neo-4 behaved similar to the natural IL-4 cytokine, although this must be examined in vivo.

Designing proteins that recapitulate the function of natural cytokines is not the end of the engineering path, as the cytokines being mimicked are therapeutically limited by their pleiotropic effects. Before the advent of de novo protein design, efforts were underway to engineer natural cytokines to improve specificity^5,19,43,44^. It is daunting to evolve a protein to preserve some functional activities while attenuating others, and although engineering natural cytokines can skew signaling activities, they do not completely eliminate native cytokine activities^5,43,45^. By contrast, de novo computationally designed cytokine mimetics have the structural and functional dexterity to redefine signaling specificity. Indeed, Neo-4 proteins are the first IL-4 agonists that show complete bias toward the type I complex. By contrast, a type I complex-biased mutein (super-4), generated from directed evolution of hIL-4, activated type II complex signaling despite its enhanced affinity for hγ_c_ and reduced affinity for hIL-13Rα1 (ref. 5). The increased specificity of Neo-4 may lead to therapeutically advantageous properties and/or reduced toxicities compared to IL-4 (refs. 2,3). IL-13 is the primary inducer of type 2 inflammation, which drives pathogenesis of multiple inflammatory diseases^2,3^. Thus IL-4-based therapeutics that maintain type I complex-mediated immune effects while avoiding type II complex-mediated inflammation could have therapeutic benefits, for instance in tissue regeneration^2^. Furthermore, the consummate bias of Neo-4 makes it an excellent tool for future studies aimed at decoupling the functional activities of type I versus type II complexes.

This study probed IL-4 biology by developing mechanistic models parametrized by IL-4 and Neo-4 signaling data. In addition to implementing a sequential model^34^, as in previous reports^5^, we designed a multivalent binding model, which allows flexibility in dimerization order^35^. Our model highlighted the discrepancy between observed signaling on primary MDMs and predicted activity based on type I:type II complex ratio. Whereas the type I complex predominates on MDMs, type I complex-biased hNeo-4 elicited attenuated STAT6 activation, and the type II complex-biased hIL-13 was fully potent on these cells. This finding suggests that additional factors beyond receptor expression levels may dictate IL-4 behavior^9^. Sensitivity analysis indicated that myeloid cells are particularly sensitive to hγ_c_ and hIL-13Rα1 levels, suggesting that these cells may use different dimerization and signaling mechanisms. This possibility is supported by evidence of an optimal hγ_c_ cluster size for signaling induction^25^ and differential dimerization dynamics for type I versus type II complexes^46^. Cytokine signaling on fibroblast and A549 cells was dependent on the γ_c_:IL-4Rα ratio rather than on absolute receptor numbers, as hNeo-4 did not activate these cells, although hγ_c_ is present. hγ_c_ signaling reportedly requires an optimal cluster size, which may not be achieved on these cells^25^. To build a more sophisticated mechanistic model, single-molecule microscopy studies could be used to better characterize cytokine–receptor binding dynamics in live cells^20^.

The thermostability of Neo-4 confers potential advantages relative to natural cytokines for biomaterials manufacturing. Cytokine delivery systems include nanoparticles, liposomes or reservoir-based formulations, but the temperature sensitivity of natural cytokines leads to protein degradation or reduced bioactivity during encapsulation or release^47^. We demonstrated that although natural cytokines can be incorporated into a 3D-printed biomaterial, our engineered de novo cytokine was far more robust to the process. hNeo-4 retained signaling when printed into two different scaffolds, and its compatibility with prolonged printing processes enables incorporation into translationally relevant large-scale biomaterials. Moreover, hNeo-4 and mNeo-4 are produced in high yield (20–40 mg liter^−1^) from *E. coli*, making these proteins more accessible and affordable than natural cytokines for biomaterials manufacturing. While de novo engineered cytokines closely mimicked the serum and protease stability properties of natural proteins, future engineering efforts could improve upon these properties. Serum half-life could also be enhanced through fusion to an Fc domain or serum albumin.

Natural cytokine pathways show extensive overlap, sharing and redundancy in receptor chain usage. This remarkable convergence can be harnessed in the context of de novo engineering to produce networks of protein mimetics from a single prototype, simplifying molecular design workflows. Together with rapidly advancing structural biology and computational prediction tools, de novo engineering efforts promise basic and translational advances in cytokine engineering.

## Online content

Any methods, additional references, Nature Portfolio reporting summaries, source data, extended data, supplementary information, acknowledgements, peer review information; details of author contributions and competing interests; and statements of data and code availability are available at https://doi.org/10.1038/s41589-023-01313-6.

## Methods

### Cell culture

Human Ramos 2G6.4C10 cells (ATCC) were cultured in RPMI-1640 (ATCC) supplemented with 10% fetal bovine serum (FBS; Hyclone) and 100 U ml^−1^ penicillin–streptomycin (pen–strep; Gibco). A549 cells (ATCC) were cultured in Ham’s F-12K (Kaighn’s) medium (Gibco) supplemented with 10% FBS and 100 U ml^−1^ pen–strep. Human dermal fibroblasts were cultured in FibroLife fibroblast serum-free complete medium (Lifeline Cell Technology). Mouse A20 cells were cultured in RPMI-1640 (ATCC) supplemented with 10% FBS, 100 U ml^−1^ pen–strep and 0.05 mM 2-mercaptoethanol (BME; Millipore). Mouse NIH/3T3 cells were cultured in DMEM (Mediatech) supplemented with 10% FBS, 2 mM l-glutamine (Gibco) and 100 U ml^−1^ pen–strep. Mouse naive T cells were cultured in TexMACS medium supplemented with 10% FBS, 100 U ml^−1^ pen–strep, 0.01% BME, 10 ng ml^−1^ IL-2 and 10 mg ml^−1^ anti-interferon-γ. Before signaling assays, Ramos and A20 cells were starved overnight in growth medium with 2% FBS. All cells were maintained at 37 °C with 5% CO_2_.

### Computational design of de novo cytokine mimetics

The ternary crystal structure of Neo-2 in complex with its receptor subunits hIL-2Rβ and hγ_c_ was aligned with the ternary crystal structure of IL-4 in complex with the type I IL-4 receptor (hIL-4Rα and hγ_c_). Fourteen amino acids in Neo-2 that constitute its interface with IL-2Rβ were mutated to match the structurally corresponding amino acids of IL-4 that constitute its interface with IL-4Rα. The resulting sequence, named hIL4s11, was cloned into the yeast surface display vector pETCON3 in frame with a C-terminal Myc tag and amplified using the MutaGene II mutagenesis kit (Invitrogen), per the manufacturer’s instructions, to yield a mutation frequency of approximately 1% per nucleotide. The resulting randomly mutagenized library was transformed into *Saccharomyces cerevisiae* EBY100 yeast and selected multiple times in succession for binding to the type I IL-4 receptor, each time under progressively more stringent binding conditions until convergence (Supplementary Table 3)^17^. Briefly, 1 × 10^8^ yeast cells expressing individual variants of the naive unselected library were resuspended in 1.2 ml of PBSA buffer (PBS (Fisher Scientific; pH 7.3) plus 0.1% bovine serum albumin (BSA; Thermo)) containing 10 nM biotinylated hIL-4Rα and incubated at 4 °C for 25 min. The yeast cells were then pelleted, washed and resuspended in 600 μl of secondary incubation buffer (557 μl of PBSA + 10 μl of streptavidin–phycoerythrin (Invitrogen) + 33 μl of FITC-conjugated Myc antibody (FITC, ICL)) and incubated at 4 °C for 15 min. The yeast cells were then pelleted, washed with 1 ml of PBSA and resuspended in 10 ml of PBSA. A negative-control sample was treated in the same way but incubated in PBSA containing no hIL-4Rα during the first incubation. The yeast cells were then sorted by FACS using a Sony SH800 cell sorter (Extended Data Fig. 2). The gate was drawn such that a very low number (0.00–0.01%) of cells from the negative-control sample would pass the gate, whereas a larger number (0.2–6.5%) of cells from the labeled population would be selected. Selected yeast cells were allowed to expand before the next sort. Biotinylated IL-4Rα (750 pM) was used for the second round of selection, and 50 nM biotinylated hγ_c_ (omitted for the negative-control sample) and 3 nM unlabeled mIL-4Rα were used for the third round of selection to allow selection of variants that were able to bind both subunits of the hIL-4Rα–hγ_c_ complex simultaneously. After expansion, 1 nM biotinylated hγ_c_ and 500 pM unlabeled mIL-4Rα were used in the final round of selection. At this point, the library was sequenced and found to be convergent on a single variant with increased affinity to the hIL-4Rα–hγ_c_ complex. This variant, named hNeo-4, was recombinantly expressed and purified from *E. coli* and characterized by SDS–PAGE and size-exclusion chromatography to confirm its solubility and monomericity.

The mIL-4 mimic was created similarly. A predicted structure of mIL-4, generated by Robetta^48^, was aligned into the ternary crystal structure alignment above, and 11 amino acids in hIL4s11 were mutated, constituting its interface with IL-4Rα that differed from those of the mIL-4 predicted structure. In addition, Cys 87 in the predicted mIL-4Rα interface, which is predicted to form a disulfide bridge with Cys 5, was mutated to alanine to prevent an unpaired cysteine in the mIL-4 mimic’s interface. The resulting sequence with 12 mutations, named mIL4s11, was randomly mutagenized and transformed into EBY100 yeast as described above. Yeast cells (5 × 10^7^) expressing individual variants of the unselected mouse library were resuspended in 600 μl of PBSA buffer containing 1 μM biotinylated tetrameric mIL-4Rα (omitted for the negative-control sample), 250 nM streptavidin–phycoerythrin and 16.6 μl of FITC and incubated at 4 °C for 25 min while slowly inverting. The yeast cells were then pelleted, washed and resuspended in 5 ml of PBSA for FACS. After allowing the selected yeast to expand, a second sort was performed with 500 nM tetrameric biotinylated mIL-4Rα, and a final sort was performed with 100 nM tetrameric biotinylated mγ_c_ and 100 nM unlabeled mIL-4Rα. The library was sequenced and found to have converged on a variant with increased affinity to the mIL-4Rα–mγ_c_ complex. This variant was recombinantly expressed and purified from *E. coli* and named mNeo-4.

### Neo-4 crystal structure

Neo-4 was crystallized at 60 mg ml^−1^ by vapor diffusion by mixing with an equal volume of well solution (3.5 M NaCl, 0.1 M sodium acetate (pH 5.1) and 5% DMSO) and equilibrating against the well solution. Crystals were cryoprotected by the addition of 5 vol of 3.4 M sodium malonate (pH 5.0) and flash-frozen in liquid nitrogen. Diffraction data were collected at APS beamline 23 ID-B at 12,000 eV. Reflections were indexed in space group H32 with cell dimensions *a* = *b* = 122.477 Å and *c* = 310.376 Å and integrated and scaled with XDS^49^ with a dmin of 2.97-Å resolution.

The structure was solved by molecular replacement in Phaser^50^. For the search model, the structure of Neo-2 (Protein Data Bank (PDB) ID 6DG6) was modified by the removal of loops and truncation of all side chains to Cβ. Eight monomers were found in the asymmetric unit, and the topology of the solution was validated by observation of electron density for missing loops and aromatic side chains in a prime-and-switch map. Initial model rebuilding was performed with Phenix autobuild^51^, followed by iterative cycles of rebuilding in Coot^52^ and reciprocal space refinement in Buster^53^, with a final refinement in Phenix using individual atomic displacement parameters and TLS and assignment of each chain to a single TLS group. NCS was initially constrained, and torsional restraints were introduced. In the final refinement, NCS limits were reduced to 5° to allow for differences observed between the chains. Crystallographic software used in this work was installed and maintained by SBGrid. Crystallographic data collection and refinement statistics are presented in Supplementary Table 10. The crystallographic model and reduced reflections have been deposited in the PDB with accession code 8DZB. Diffraction images have been deposited in the SBGrid Data Bank with digital object identifier 959. Protein visualization was performed using PyMOL 2.5.

### Protein structure modeling using AlphaFold2

The predicted model of hNeo-4 in complex with IL-4Rα and γ_c_ was calculated using Alphafold2-multimer-v2 on the ColabFold server without template information^54^. The top five models were relaxed using Amber^55^. Models with egregious errors (for example, γ_c_ in a physically disallowed upside-down conformation) were excluded from analysis.

### Protein expression and purification

Genes encoding the designed protein sequences, including hNeo-4, mNeo-4 and Neo-2, were synthesized and cloned into pET-29b(+) *E. coli* plasmid expression vectors (GenScript). For all the designed proteins, an N-terminal tag with the sequence MGSHHHHHHGSGSENLYFQGS was added immediately before the sequence of the designed protein. Plasmids were transformed into chemically competent *E. coli* Lemo21 cells (NEB), and protein expression was performed using Terrific Broth and M salts. Cultures were grown at 37 °C until the absorbance at 600 nm reached approximately 0.8, expression was induced with 1 mM isopropyl-β-D-thiogalactopyranoside, and the temperature was lowered to 18 °C. After expression for approximately 18 h, cells were collected and lysed with a Microfluidics M110P microfluidizer at 18,000 psi. The soluble fraction was clarified by centrifugation at 24,000*g* for 20 min and purified by immobilized metal affinity chromatography (Qiagen) followed by FPLC size-exclusion chromatography (Superdex 75 10/300 GL, GE Healthcare). The purified proteins were analyzed by SDS–PAGE and MS. Fractions containing monomeric protein were pooled and concentrated, the concentration was measured by absorbance at 280 nm (Nanodrop), and samples were snap-frozen in liquid nitrogen and stored at −80 °C. Proteins used in animal studies were further purified to remove endotoxins using NoEndo high-capacity spin columns (Protein Ark). The endotoxin levels were measured with an Endosafe LAL cartridge (PTS201F, 0.1 EU ml^−1^ sensitivity; Charles River) in an Endosafe nexgen-MCS system (Charles River).

hγ_c_ (amino acids 34–232) or mγ_c_ (amino acids 34–232) were expressed and purified using a baculovirus expression system^56^. The IL-4Rα ectodomain (amino acids 1–202) was expressed and purified using another baculovirus expression system^57^. For protein biotinylation, hIL-4Rα, mIL-4Rα or mγ_c_ was biotinylated in vitro using the soluble BirA ligase enzyme in 0.5 mM bicine (pH 8.3), 100 mM ATP, 100 mM magnesium acetate and 500 mM biotin (Sigma). All proteins contained C-terminal hexahistidine tags, were isolated by nickel chromatography and were further purified to >98% homogeneity by size-exclusion chromatography on a Superdex 200 column (GE Healthcare) equilibrated in 10 mM HEPES (pH 7.3) and 150 mM NaCl.

Super-4 was purified using the BacuVance baculovirus expression system (Genscript). The super-4 sequence was synthesized and cloned into the pAcGP67A vector in frame with an N-terminal gp67 signal sequence and C-terminal hexahistidine tag. *Spodoptera frugiperda* (Sf9) cells were transduced with the pAcGP67A vector for preparing the baculovirus stocks. Super-4 was then purified from the baculovirus-transfected Sf9 cells. Proteins were expressed and captured from HiFive supernatants after 3–4 d by nickel agarose resin.

### Circular dichroism

Far-ultraviolet CD measurements were performed with an AVIV spectrometer model 420 in PBS (pH 7.4) in a 1-mm path length cuvette with a protein concentration of ~0.20 mg ml^−1^. Temperature melts were performed from 25 to 95 °C, and absorption signal was monitored at 222 nm (steps of 2 °C min^−1^, 30 s of equilibration by step). Wavelength scans (195–260 nm) were collected at 25 °C and 95 °C and again at 25 °C after fast refolding (~5 min).

### Biolayer interferometry

Binding data were collected in an Octet RED96 (ForteBio). To measure the binding affinity of hIL-4 or hNeo-4 to hIL-4Rα, biotinylated hIL-4Rα was immobilized on streptavidin-coated biosensors (SA ForteBio) at 5 μg ml^–1^ in binding buffer (HBS-EP+ (Cytiva) and 0.5% blotting-grade blocker non-fat dry milk (Bio-Rad, 1706404XTU)) until a signal of 0.5 nm was reached. The biosensors were then exposed to threefold serial dilutions of hIL-4 (Acro Biosystems, IL-4-H4218) or hNeo-4 starting at 200 nM for 900 s to measure association, and dissociation was measured exposing the sensors to binding buffer for 1,200 s. Similarly, to quantify the binding to the IL-4 type I or type II complex, biotinylated hγ_c_ or polyhistidine-tagged hIL-13Rα1 (Acro Biosystems, IL1-H5224) were immobilized on streptavidin-coated biosensors (ForteBio) or His1K biosensors (ForteBio), respectively. Association was measured by exposing the immobilized receptors to threefold serial dilutions of hIL-4 (Acro Biosystems, IL-4-H4218) or hNeo-4 starting at 333 nM for type I receptor binding and starting at 1 μM for type II receptor binding in the presence of twofold molar excess of soluble of hIL-4Rα-His (Acro Biosystems, ILR-H5221). In addition, the binding specificity of hNeo-4 to the IL-4 receptors was evaluated by immobilizing biotinylated hIL-2Rβ (Acro Biosystems, ILB-H82E3) or hIL-2Rβ–hγ_c_ dimer (Acro Biosystems, CD2-H5221 and ILG-H85E8) on streptavidin-coated biosensors (ForteBio) as described above. For hIL-2Rβ binding, biosensors were exposed to threefold serial dilutions of Neo-2 starting at 60 nM or hNeo-4 starting at 100 nM, respectively. For hIL-2Rβ–hγ_c_ binding, biosensors were exposed to threefold serial dilutions of Neo-2 starting at 33 nM or hNeo-4 starting at 300 nM, respectively.

To measure the binding affinity of mIL-4 or mNeo-4 to mIL-4Rα, biotinylated mIL-4Rα was immobilized on streptavidin-coated biosensors as described above and exposed to threefold serial dilutions of mIL-4 (R&D Systems, 404-ML/CF) starting at 67 nM or mNeo-4 starting at 270 nM. To evaluate the binding to the mIL-4 type I complex, biotinylated mγ_c_ was immobilized on streptavidin-coated biosensors exposed to threefold serial dilutions of mIL-4 (R&D Systems, 404-ML/CF) starting at 650 nM or mNeo-4 starting at 750 nM in the presence of twofold molar excess of soluble polyhistidine-tagged mIL-4Rα.

Data were processed using ForteBio Data Analysis Software version 9.0.0.10., and the parameters were reported with standard error. All raw data were fitted using a 1:1 Langmuir binding model. Equilibrium titration curve fitting and determination of the *K*_d_ values with standard error was implemented using a first-order logistic model using the ‘[Agonist] vs. normalized response—variable slope’ model in Prism software (GraphPad). All data are summarized in Supplementary Table 5.

### Human monocyte isolation and monocyte-derived macrophage culture

Human peripheral blood mononuclear cells (PBMCs) were obtained from leukopaks. Briefly, whole blood from leukopaks was mixed with 1× PBS, and Ficoll-Hypaque solution (Cytiva) was carefully laid underneath of the blood and PBS mixture. Cells were centrifuged at 400*g* for 30 min with no break. After the centrifugation, cells in the buffy coat were collected and lysed with ACK lysis buffer (Gibco) on ice for 5 min and washed with excess amounts of RPMI (Gibco) to obtain PBMCs. CD14^+^ monocytes were isolated (>95% purity; Extended Data Fig. 4g) from the PBMCs by using CD14 MicroBeads (Miltenyi Biotec) following the manufacturer’s instructions. One hundred million cells were incubated with 200 μl of CD14 MicroBeads for 15 min at 4 °C, washed with MACS buffer and loaded onto an LS separation column (Miltenyi Biotec). CD14^+^ cells were eluted from the column after three washes with MACS buffer. The purity of the monocytes was evaluated via flow cytometry.

Human macrophages were differentiated from the monocytes. Briefly, CD14^+^ monocytes were cultured in RPMI-1640 supplemented with 10% FBS, 100 U ml^−1^ pen–strep and 50 ng ml^−1^ human macrophage colony-stimulating factor (M-CSF; BioLegend) at 37 °C with 5% CO_2_ for 7 d. Medium was refreshed on day 4, and before signaling assays, human macrophages were starved in growth medium overnight with 2% FBS.

### Mouse bone marrow-derived macrophage culture

Mouse BMDMs were differentiated from monocytes in the bone marrow. Briefly, femurs and tibias were dissected from both legs of a C57BL/6 mouse, and one end of each bone was cut open with a pair of scissors. Bone marrow was obtained by centrifuging bones with open ends at 2,000*g* for 30 s. The collected bone marrow was resuspended and cultured in DMEM supplemented with 10% FBS, 100 U ml^−1^ pen–strep, 2 mM l-glutamine and 100 ng ml^−1^ mouse M-CSF (BioLegend) for 7 d at 37 °C with 5% CO_2_ to obtain mouse macrophages. Medium was refreshed on day 4, and before signaling assays, mouse macrophages were starved in growth medium overnight with 2% FBS.

### Cell signaling assays

Ramos cells (starved), human primary monocytes, human primary macrophages (starved), human primary fibroblasts or A549 cells were plated in a 96-well plate (2 × 10^5^ cells per well) and incubated in serial dilutions of hIL-4 (R&D Systems), hIL-13 (R&D Systems) or hNeo-4 for 20 min at 37 °C. For the signaling assays with hIL-13Rα1 neutralizing antibody, cells were incubated with serial dilutions of protein treatments with goat anti-human IL-13Rα1 polyclonal antibody (R&D Systems) at a saturating concentration of 200 nM (Extended Data Fig. 7). Cells were then fixed with 1.6% paraformaldehyde, permeabilized with methanol and incubated with a 1:100 dilution of Alexa Fluor 647-conjugated antibody to pSTAT6 (Cell Signaling Technology, clone D8S9Y) in PBSA for 2 h at room temperature. Cells were then washed twice in PBSA and analyzed on a CytoFLEX flow cytometer (Beckman Coulter).

Similarly, A20 cells (starved), mouse primary macrophages (starved) or NIH/3T3 cells were plated in a 96-well plate (2 × 10^5^ cells per well) and incubated in serial dilutions of mIL-4 (BioLegend) or mNeo-4 for 20 min at 37 °C. Cells were then processed and analyzed as described above. For all experiments, background-subtracted fluorescence measurements were normalized to hIL-4-treated samples at a saturating concentration. Curves were fitted to a first-order logistic model, and EC_50_ values were calculated using Prism software (GraphPad). Experiments were conducted with at least two technical repeats and performed at least twice with similar results.

For detection of minor activation of STAT1, STAT3 and STAT5, samples were fluorescently barcoded. In brief, following paraformaldehyde fixation, cells were resuspended in 100 μl of methanol and permeabilized for 30 min on ice. Then, various combinations and concentrations (fivefold dilutions from 2 μg ml^−1^) of Alexa Fluor 488 TFP (Thermo), PacificBlue NHS (Thermo) and PacificOrange NHS (Thermo) were added with PBS to a final volume of 180 μl. Samples were incubated for 30 min at 4 °C. Samples were then thoroughly washed with TBS to quench excess dye. Samples were pooled into a single tube using PBSA and split into separate wells for staining. Samples were then stained with Alexa Fluor 647-conjugated anti-pSTAT1 (Cell Signaling, Clone 58D6), anti-pSTAT3 (Cell Signaling, Clone D3A7) or anti-pSTAT5 (BD, Clone 47) for 2 h at room temperature and analyzed on a CytoFLEX flow cytometer (Beckman Coulter), as described above.

### Human monocyte polarization

One million human monocytes were stimulated with 100 nM hIL-4 (R&D Systems) or hNeo-4 for 24 h at 37 °C. Cells were washed and lysed with TriReagent and flash-frozen to store in a −80 °C freezer. mRNA was isolated using a Direct-zol RNA microprep kit (R2061, ZYMO Research). All RT–qPCRs were performed using TaqMan gene expression master mix (Applied Biosystems) and probes according to the manufacturer’s instructions. Briefly, 200 ng of mRNA was used to synthesize cDNA using Superscript IV VILO master mix (Thermo Fisher Scientific) using the manufacturer’s guidelines and a C1000 Touch thermocycler (Bio-Rad). Five nanograms of cDNA was then used for each well of the RT–qPCR. All RT–qPCRs were performed on the StepOne Plus real-time PCR system and software (Applied Biosystems, Thermo Fisher Scientific) as TaqMan single-plex FAM-MGB assays, using manufacturer-recommended settings for quantitative and relative expression. TaqMan gene expression master mix (Applied Biosystems) and probes were added to all RT–qPCRs according to manufacturer’s instructions. *GAPDH* was used as an endogenous control. Samples were normalized to PBS-treated controls. All RT–qPCR data were analyzed using the Livak method^58^, wherein fold change (FC) values were calculated by the standard 2^−ΔΔ***C***^_***t***_ method. The log_2_ (FC) values are represented by the means, with error bars representing the standard deviation (*n* = 4). Assay IDs of probes used in the RT–qPCRs are included in Supplementary Table 11.

### Mouse macrophage polarization

One million mouse BMDMs per well were seeded in six-well plates. On day 7 of macrophage culture, medium was refreshed without M-CSF. Cells were stimulated with 1.5 nM mIL-4 (BioLegend) or 1.5 mM mNeo-4 for 24 h. Cells were then washed with PBS and directly lysed with 0.5 ml of buffer RLT Plus (Thermo Fisher Scientific) with 1% (vol/vol) BME. Cells were vortexed for 5 s in RLT buffer, flash-frozen and stored in a freezer at −80 °C. mRNA was isolated using a Qiagen RNeasy Plus kit with gDNA eliminator columns. All RT–qPCRs were performed similar to as described above. Forty nanograms of cDNA was used for each well of RT–qPCR. *Rer1* was used as an endogenous control. Samples were normalized to PBS-treated controls. All RT–qPCR data were analyzed as described above. The log_2_ (FC) values are represented by the means, with error bars representing the standard deviation (*n* = 4). Assay IDs of probes used in the RT–qPCRs are included in Supplementary Table 11.

### Primary naive T cell polarization

Naive T cells were isolated from the spleens of IL-4-IRES-eGFP (4GET) reporter mice using mouse naive CD4^+^ T cell isolation kits (Miltenyi Biotec) and cultured in complete TexMACS medium. Cells were activated via CD3ε–biotin and CD28–biotin beads (Miltenyi Biotec) and treated with eightfold serial dilutions of mIL-4 or mNeo-4 starting at 125 nM. Cells were cultured for 5 d and collected for GFP expression analysis on a CytoFLEX flow cytometer (Beckman Coulter). EC_50_ values were calculated using first-order logistic fitting models in GraphPad Prism after subtraction of the mean fluorescence intensity (MFI) of unstimulated cells. Experiments were conducted in singlet and performed twice with similar results.

### RNA-seq library preparation

Human CD14^+^ monocytes were purified from buffy coats from healthy donors at the NIH Blood Bank using human CD14 microbeads, as described above, and passing over LS columns twice. For RNA-seq experiments, monocytes were obtained from two healthy donors. From each donor, 6 × 10^5^ purified monocytes were cultured in 0.5 ml of complete RPMI-1640 in 48-well plates and stimulated for 24 h with 100 nM hIL-4, hIL-13 or hNeo-4 or PBS. Total RNA was isolated using the Direct-zol RNA Microprep kit, and RNA-seq libraries were prepared using 120 ng of total RNA and a Kapa mRNA HyperPrep kit (KK8580, Kapa Biosystems). Each library was indexed using NEXTflex DNA Barcodes-24 (BIOO Scientific) and amplified by PCR. PCR-amplified libraries between 250 and 400 base pairs long were recovered using 2% E-Gel precast gels (Thermo Fisher), purified with a Zymoclean gel DNA recovery kit (Zymo Research) and quantified by Qubit 3 fluorometer (Thermo Fisher). Equal amounts of the indexed libraries were then pooled and sequenced with an Illumina NovaSeq platform at the National Heart, Lung, and Blood Institute (NHLBI) DNA Sequencing Core.

### RNA-seq data analysis

Sequenced reads (50 base pairs, single end) were obtained with the Illumina CASAVA pipeline and mapped to the human genome hg38 (GRCh38, December 2013) using Bowtie2 and Tophat2. Only uniquely mapped reads were retained. Raw counts that fell on exons of each gene were calculated and normalized by using reads per kilobase per million mapped reads. The R Bioconductor package ‘edgeR’ was used to identify differentially expressed genes among cells treated with hIL-4, hIL-13 or hNeo-4. Venn diagrams were generated using venndiagram. tk^59^. Gene expression heat maps were generated with the R package ‘pheatmap’.

### RT–qPCR validation of gene expression in RNA-seq

To validate the differential gene expression profiles shown in the hNeo-4-treated group, mRNA samples from the monocytes of two additional healthy donors were prepared and processed using the exact procedure described above. One hundred and eighty nanograms of mRNA from each sample was used to synthesize cDNA, and 5 ng of cDNA was used for each RT–qPCR well as described above. *GAPDH* was used as an endogenous control. Assay IDs of probes used in the RT–qPCRs are included in Supplementary Table 11. Samples were normalized to PBS-treated controls. All RT–qPCR data were analyzed using the Livak method^58^. The log_2_ (FC) values are represented by the means, with error bars representing the standard deviation.

### In vitro toxicity studies

Mouse CD4^+^ T cells were isolated from the spleens of female C57BL/6 and activated as described above in T_H_2 polarization studies. Samples were treated with dilutions of mIL-4 or mNeo-4 for 1, 3 and 5 d. Human CD4^+^ T cells were isolated from buffy coats using a human CD4^+^ T cell isolation kit (Miltenyi). Isolated human CD4^+^ T cells were cultured in RPMI containing 10% FBS and 15 ng ml^−1^ IL-2 (Peprotech) and activated with anti-human CD3/CD28 Dynabeads (Thermo). Samples were treated with serial dilutions of IL-4 or Neo-4 for 1, 3 and 5 d. Samples were stained with the SYTOX red viability dye to quantify expansion and viability (Thermo) and were simultaneously stained with CellROX green (Thermo) for reactive oxygen species detection, according to the manufacturer’s protocols. Samples were then analyzed on a CytoFLEX flow cytometer.

For LDH activity measurements, human CD14^+^ monocytes and CD4^+^ T cells were isolated as described above. Monocytes were treated with dilutions of both hIL-4 and hNeo-4 without any growth factor in RPMI containing 10% FBS. CD4^+^ T cells were cultured and activated as described above and treated with dilutions of hIL-4 or hNeo-4. Supernatants were collected on days 1, 3 and 5 and assayed for LDH activity using the CyQuant LDH cytotoxicity assay (Thermo). Samples were then analyzed on a Synergy 2 multimode microplate reader (Biotek).

### mNeo-4 evaluation in the volumetric muscle loss mouse model

The in vivo functionality of mNeo-4 was characterized using the volumetric muscle mouse model^33^. Briefly, 6- to 8-week-old female C57BL/6 ( Jackson Laboratories) mice were anesthetized with 3.0% isoflurane and maintained under 2.5% isoflurane. Hair was removed from the lower extremities with an electric razor (Oster). After ethanol sterilization of the surrounding skin, a 1.5-cm incision was created between the knee and hip joint to access the quadriceps femoris muscle. A 3 mm × 3 mm × 3 mm defect was created in the quadriceps femoris muscle group using surgical scissors. On day 0, the resulting bilateral defects on each mouse were filled with 100 μl of protein treatment. Starting on day 1, mice were subcutaneously injected daily for 3 d with the same dose of protein treatments. On day 3, the mice were killed, and their entire quadriceps femoris muscle was removed by cutting from the knee joint along the femur to the hip joint.

Because there were no reported data on the VML model using IL-4 as the sole systemic treatment, we first tested 1.5 μg per mouse per d as a conventional dose of mIL-4 in a preliminary study (*n* = 4; Extended Data Fig. 6). After confirming that the effective dose for mIL-4 is 1.5 μg per mouse per d, we conducted another pilot study with the same surgical process and dosing regimen to compare the effect of mIL-4 (*n* = 4) and mNeo-4 (*n* = 4) at 1.5 μg per mouse per d. Following the pilot study, we conducted a more thorough study with 1.5 μg of mIL-4 (*n* = 5), 0.03 μg of mNeo-4 (*n* = 5), 1.57 μg of mNeo-4 (*n* = 5), 70.8 μg of mNeo-4 (*n* = 4) or PBS treatment (*n* = 5). Again, the same surgical process and dosing regimen as in the pilot study was used. Experiments were performed twice with similar results.

All procedures conducted were approved through the Johns Hopkins University Animal Care and Use Committee, in accordance with the NIH Guide for the Care and Use of Laboratory Animals. All mice were maintained in housing with a 12-h light/12-h dark cycle, with 30–70% relative humidity and a temperature of 18–26 °C.

### RT–qPCR for gene expression in the volumetric muscle loss model

Immediately after death, collected skeletal muscle tissues and spleens were placed into 1 ml of TRIzol reagent (Thermo Fisher Scientific), flash-frozen and stored in a freezer at −80 °C. All tissue samples were homogenized using a Bead Ruptor 12 (OMNI International) using the highest speed setting for 45 s with 2.8-mm ceramic beads (OMNI International). Enriched mRNA was isolated from whole tissue using TRIzol reagent and a Qiagen RNeasy Plus kit with gDNA eliminator columns. All RT–qPCRs were performed similar to as described above. Enriched mRNA (2.5 μg) was used to synthesize cDNA, and 100 ng of cDNA was used in each RT–qPCR well. For tissue samples, both *Rer1* and *Hprt* were used as endogenous controls, with samples normalized to the most stable endogenous control. Samples were normalized to PBS controls. All RT–qPCR data were analyzed using the Livak method^58^ and further verified by analysis using the Applied Biosystems relative quantification online software (Thermo Fisher Scientific, version 2020.2.1-Q2-20-build4). The log_2_ (FC) values are represented by the geometric means, with error bars representing the geometric standard deviation. Assay IDs of probes used in the RT–qPCRs are included in Supplementary Table 11.

### NanoString analysis for mNeo-4

Muscle samples from 12 mice in the previously described VML mouse study, including 4 PBS-treated mice, 4 mIL-4-treated mice (1.5 μg per mouse per d) and 4 mNeo-4-treated mice (70.8 μg per mouse per d), were used to evaluate the gene expression profiles of mIL-4 and mNeo-4 treatments. NanoString differential expression assays (NanoString Technologies) were performed according to the manufacturer’s recommendations. After mRNA isolation using the RNeasy Plus mini-kit system, 100 ng of mRNA from each muscle sample was used for hybridization reactions. The mouse myeloid innate immunity codeset (XT-CSO-MIP1-12, NanoString) was used for probe hybridization and annotations. The probe hybridization reaction was set to 20 h at 65 °C in a C1000 Touch thermocycler (Bio-Rad). The assay was performed using the nCounter MAX analysis system. The Prep Station was performed on the high-sensitivity protocol, and the Digital Analyzer imaging protocol was set to max resolution. Results were analyzed using nSolver Advanced Analysis (version 3.0, NanoString). The top 20 reference genes were selected by the software and used for normalization. The default threshold of 20 counts was used for the analysis. False discovery rate-adjusted *P* values were determined for each gene using the Benjamini–Yekutieli method.

### Receptor staining and quantification

The surface expression levels of IL-4Rα, γ_c_ and IL-13Rα1 on each human or mouse cell type were quantified using Quantum Simply Cellular anti-mouse IgG beads and anti-rat IgG beads (Bangs Laboratories), according to the manufacturer’s protocol. Briefly, standard beads were incubated with a 1:20 dilution of APC-conjugated mouse anti-human IL-4Rα (BioLegend, clone G077F6), APC-conjugated rat anti-human γ_c_ (BioLegend, clone TUGh4), APC-conjugated mouse anti-human IL-13Rα1 (BioLegend, clone SS12B), APC-conjugated rat anti-mouse IL-4Rα (BioLegend, clone I015F8), PE-conjugated rat anti-mouse γ_c_ (BioLegend, clone TUGm2) or PE-conjugated rat anti-mouse IL-13Rα1 (Invitrogen, clone 13MOKA) in PBSA for 30 min at 4 °C. The beads were then washed twice, resuspended in PBSA and analyzed on a CytoFLEX flow cytometer (Beckman Coulter). Data were analyzed using FlowJo v10 to generate a standard curve. Each type of cell was resuspended in PBSA and seeded into 96-well plates with 2 × 10^5^ cells per well. Cells were incubated with a 1:20 dilution of human Fc blocking (BioLegend) and human monocytes blocking (BioLegend) mixture or mouse Fc blocking mixture (BioLegend) for 10 min on ice. Immediately after blocking, cells were incubated with an antibody solution containing either anti-human or mouse IL-4Rα, γ_c_, or IL-13Rα1 in PBSA, as described above. Cells were washed twice, resuspended in PBSA and analyzed via flow cytometry at the same time with standard beads. MFI values were compared to the generated calibration curve to determine the receptor expression levels. Experiments were performed at least twice with similar results.

### IL-4/IL-13 signaling pathway modeling using multivalent and sequential binding models

The multivalent and sequential binding models used to predict cell-type-specific signaling responses to IL-4, Neo-4 and IL-13 were formulated^34,35^. In the multivalent model, each IL-4 molecule was assumed to bind to one free IL-4Rα and one of either γ_c_ or IL-13Rα1, whereas, in the sequential model, each IL-4 molecule and mimetic was assumed to first bind IL-4Rα and subsequently one of either γ_c_ or IL-13Rα. IL-13 was assumed to first bind to IL-13Rα and subsequently IL-4Rα. Both models were fit to experimentally gathered pSTAT6 signaling data of human cell lines (Ramos B cells, primary monocytes, MDMs, primary fibroblasts and A549 cells) and mouse cell lines (NIH/3T3, BMDMs and A20 cells). In both models, the affinities with which each ligand interacted with each receptor were fit using least squares fitting. In the multivalent model, subsequent ligand–receptor binding interactions were modeled as proceeding with an association constant proportional to the free receptor abundance and the optimized affinity of receptor–ligand interaction multiplied by the crosslinking constant, *K*_x_*. A single *K*_x_* value was fit for all experiments and cell types. In both models, predicted pSTAT6 was determined by calculating the total amount of predicted type I and type II signaling complexes (ligand–IL-4Rα–γ_c_ and ligand–IL-4Rα–IL-13Rα1, respectively). The amount of pSTAT6 predicted was normalized to the maximum pSTAT6 predicted on a per cell-type basis. The abundance of each receptor subunit on each cell type was experimentally determined as described above.

### Cell signaling response principal-component analysis

A PCA was performed on the pSTAT6 signaling response of human cells to IL-4, Neo-4 and IL-13 by calculating the area under the curve of each cell line’s normalized responses to each of these ligands. Area under the curve was calculated by fitting a Hill curve to each cell’s response and determining the integral of this curve from the smallest dose at which any response in any cell was measured to the largest. All experimental replicates were individually included in the analysis. The signaling responses were arranged in a matrix format, and a PCA with two components was performed. Cell lines with incomplete ligand response data (lines only stimulated with one or two of the three ligands) were excluded from the analysis.

### Thermal stability assay

hIL-4 and hNeo-4 in PBS solution were heated at 95 °C for 15, 30, 60, 120 and 180 min in a thermocycler (Bio-Rad) before the signaling assay. Serial dilutions of hIL-4 and hNeo-4 were made using the heated and unheated protein solutions. Human Ramos cells (2 × 10^5^ per well) were seeded in a 96-well plate and stimulated with serial dilutions of the protein treatments for 20 min. Similarly, mIL-4 and mNeo-4 were also tested by heating at 95 °C for 60 min. Mouse BMDMs (2 × 10^5^) were stimulated with serial dilutions of heated and unheated mIL-4 and mNeo-4 solution. After stimulation, cells were fixed, permeabilized, stained and analyzed as described above in the signaling assay section. EC_50_ values were calculated using a first-order logistic fitting model in GraphPad Prism after subtraction of the MFI of the unstimulated cells and normalization to the saturation signal intensity. Experiments were conducted in at least duplicate and were performed twice with similar results.

### Hot-melt extrusion and three-dimensional printing

hNeo-4 was first lyophilized in PBS. To validate the bioactivity of lyophilized hNeo-4, it was reconstituted in PBS and used to stimulate Ramos cells. The bioactivity of lyophilized hNeo-4 was verified to not be compromised because it showed highly comparable EC_50_ values as hNeo-4 before lyophilization (Extended Data Fig. 9f). To make 3D-printed scaffolds, we used a two-step process involving HME to create filaments, followed by fused deposition modeling, a type of 3D printing. We created two PCL filaments, one with 10 g of PCL powder (molecular weight of 50,000 Da; 25090–500) and another with 10 g of PCL thoroughly mixed with 600 μg of hNeo-4 lyophilized in PBS (PCL + Neo-4). HME was performed on a Laboratory Mixing Extruder at 80 °C to make each filament at a diameter of 1.6 mm. For 3D printing, a Lulzbot Mini v1.0 (Fargo Additive Manufacturing Equipment 3D) with a 0.5-mm nozzle and glass print bed was used. Scaffold designs were customized to the shape of a 96-well plate, with a 6.2-mm diameter, 0.4-mm height and 0.2-mm grid infill. PCL or PCL + Neo-4 scaffolds were printed at 130 °C, with a print speed of 2.5 mm s^−1^ and 0.2-mm layer height. Final PCL + Neo-4 or PCL scaffold weights were 8.6 ± 0.4 mg.

### Direct powder extrusion and three-dimensional printing

Direct powder extrusion 3D printing was performed using the thermoplastic extrusion unit of a CELLINK BIO X6 bioprinter. A mixture of hIL-4 (Peprotech, 200–04) or hNeo-4 and a blend of PCL and PLGA (Resomer RG 753H, Sigma Aldrich, 769819) was prepared in a ratio of 0.00033:1:1 (wt/wt/wt). The mixture was fed into the heating chamber of the printer and heated to 150 °C. The cytokine/polymer blend was heated for 10 min, 30 min, 1 h, 2 h and 3 h and extruded through a 0.4-mm-diameter nozzle onto a glass print bed maintained at room temperature. A pressure of 30–50 kPa was applied pneumatically, and a print speed of 5 mm s^−1^ was used to achieve the desired print pattern.

### Protein release assay

Scaffolds were incubated in 50 μl of PBS in a 96-well flat-bottom plate at 37 °C. The supernatant was collected and replaced with 50 μl of fresh PBS at various time points. The amount of released protein was quantified using a NanoOrange protein quantitation kit (Thermo Fisher, N6666) following the manufacturer’s protocol. Briefly, 1× working solution was made by diluting protein quantitation diluent 10-fold and NanoOrange reagent 500-fold in water. To generate a standard curve, a twofold serial dilution of hNeo-4 from 8 μg ml^−1^ to 0.5 μg ml^−1^ was prepared in PBS. Then, 20 μl of each standard or collected supernatant sample was mixed with 80 μl of 1× working solution, heated at 95 °C for 10 min and allowed to cool back to room temperature for 20 min protected from light. All samples were analyzed on a Synergy 2 multimode microplate reader (Biotek). Readouts from scaffolds lacking any cytokine were used as background and were subtracted from the readouts of samples when calculating the amount of released hNeo-4 or hIL-4. The experiments were conducted in triplicate and performed twice with similar results.

To estimate the amount of released hNeo-4 used in signaling assays with PCL-only scaffolds, the PCL with or without Neo-4 scaffold was incubated in 50 μl of PBS in a 96-well flat-bottom plate at 4 °C for 16 h. The supernatant was collected, processed and analyzed using the same procedure described above. The experiments were conducted in triplicate and performed twice with similar results. On average, 3.4 ng of hNeo-4 was released in 50 μl of PBS during the incubation time (Extended Data Fig. 9i).

### Validation of hNeo-4 bioactivity after three-dimensional-printing process

PCL + Neo-4 or PCL scaffolds were individually incubated in 50 μl of Ramos starvation medium overnight. The supernatant and scaffold in each well were collected separately and added to individual 50-ml conical tubes. Different volumes of medium were added to conical tubes as indicated in Supplementary Table 12 so that the supernatant or the scaffold was diluted in a 4.5-fold serial dilution. Ramos cells (1 × 10^6^) were added to each conical tube and incubated at 37 °C for 30 min. Cells were then transferred to a 96-well, deep-well plate and fixed and permeabilized as described above for other signaling assays. Cells were stained with Alexa Fluor 647-conjugated anti-pSTAT6 analyzed on a CytoFLEX flow cytometer (Beckman Coulter). The concentration of hNeo-4 in the scaffold was estimated by the weight of the scaffold and the hNeo-4-to-PCL ratio (600 μg to 10 g) when manufacturing the 3D-printing filament. The concentration of hNeo-4 in the supernatant was estimated with an additional releasing factor of 0.67% (mean percentage of released hNeo-4 at 4 °C for 16 h), calculated based on the data shown in Extended Data Fig. 9j.

For PCL:PLGA blend scaffold studies, scaffolds were incubated in 100 μl of Ramos starvation medium or PBS for 48 h at 37 °C. PBS-treated scaffolds were subsequently used for determining protein concentration as described above. Supernatants were then titrated down in fivefold serial dilutions and used for signaling studies on Ramos cells in a 96-well plate as described above. To compare activity retention, the concentration required to achieve 10% maximal IL-4-induced STAT6 activation ([C]_10%_) across the whole experiment was interpolated based on the fit sigmoidal curves. The ratio of [C]_10%_ for the untreated cytokine relative to that of the printed cytokine (untreated/treated) was then calculated as a measure of cytokine activity retention.

### Protease stability characterization

Protease stability assays were performed to further characterize the stability of de novo designed proteins. In brief, sequencing-grade trypsin and chymotrypsin (Sigma) were reconstituted to 500 μg ml^−1^ in 1 mM HCl, and sequencing-grade pepsin (Sigma) was reconstituted to 500 μg ml^−1^ in MilliQ water (pH 4.5). For each condition, 2 μg of mIL-4, mNeo-4, hIL-4 or hNeo-4 was incubated with 1 μg of protease in a final volume of 20 μl of PBS. Pepsin digestion samples contained one-twentieth volume of 1 M HCl. Samples were incubated for 37 °C for 30, 60 and 120 min. Digestions were neutralized by adding one-fifth volume of SDS reducing buffer and boiling for 10 min, with the addition of 1 μl of NaOH for pepsin-digested samples. Samples were then subjected to SDS–PAGE analysis.

### Serum stability characterization

Protein solutions (2 mg ml^−1^) were mixed with equal volumes of corresponding serum to make protein samples at a final concentration of 1 mg ml^−1^ for each test. hIL-4 (Acro Biosystems, IL4-H52H9) or hNeo-4 was incubated with human serum (Sigma Aldrich, H6914) for up to 4 h at 37 °C. Similarly, mIL-4 (Acro Biosystems, IL4-M52H5) or mNeo-4 was incubated with mouse serum (Sigma Aldrich, S7273) for up to 4 h at 37 °C. The incubated protein samples were heated at 95 °C for 10 min with Laemli SDS buffer (Bio-Rad, 1610747) and BME (Bio-Rad, 1610710). The samples were then run on Any kD Mini-PROTEAN TGX Stain-Free protein gels (Bio-Rad, 4568124) with Tris/glycine/SDS running buffer (Bio-Rad, 1610732), along with western blotting protein ladder (Licor, 926–98000). The gels were blotted onto nitrocellulose membranes, which were then blocked with blocking buffer (TBS with 5% (wt/vol) blotting-grade blocker non-fat dry milk; Bio-Rad, 1706404XTU). Cytokines were detected by using a monoclonal antibody to 6×His with horseradish peroxidase (Invitrogen, MA1-21315-HRP) and luminated with Clarity Western ECL substrate (Bio-Rad, 1705060). Gels were imaged with a ChemiDoc XRS+ gel imager (Bio-Rad) using chemiluminescence high-resolution exposure of up to 120 s.

### Reporting summary

Further information on research design is available in the Nature Portfolio Reporting Summary linked to this article.

## Extended Data

## Supplementary Material

Suppl Info

Source data fig 2

source data fig 4

source data extended data fig 1

source data extended fig 4

source data fig 6

## Data Availability

The original experimental data that support the findings of this work are available from the corresponding authors upon request. Atomic coordinates and structure factors for the reported hNeo-4 crystal structure have been deposited with the PDB under accession code 8DZB. Diffraction images of hNeo-4 have been deposited in the SBGrid Data Bank with digital object identifier 959. The Neo-2 crystallographic structure used in this study can be accessed at the PDB database under accession code 6DG5. Plasmids encoding the proteins described in this article are available from the corresponding authors upon request. Source data are provided with this paper.
