# Supplementary material for: Design of cell-type-specific hyperstable IL-4 mimetics via modular de novo scaffolds
Source: Nat Chem Biol. Author manuscript; Available in PMC 2023 Dec 5. (PMC10697138; doi:10.1038/s41589-023-01313-6)
Supplement: Suppl Info [file NIHMS1917493-supplement-Suppl_Info.pdf]

nature chemical biology

Article

https://doi.org/10.1038/s41589-023-01313-6

Design of cell-type-specific hyperstable IL-4 mimetics via modular de novo scaffolds

In the format provided by the authors and unedited

Supplementary Information

Supplementary Figure 1
Supplementary Tables 1-12

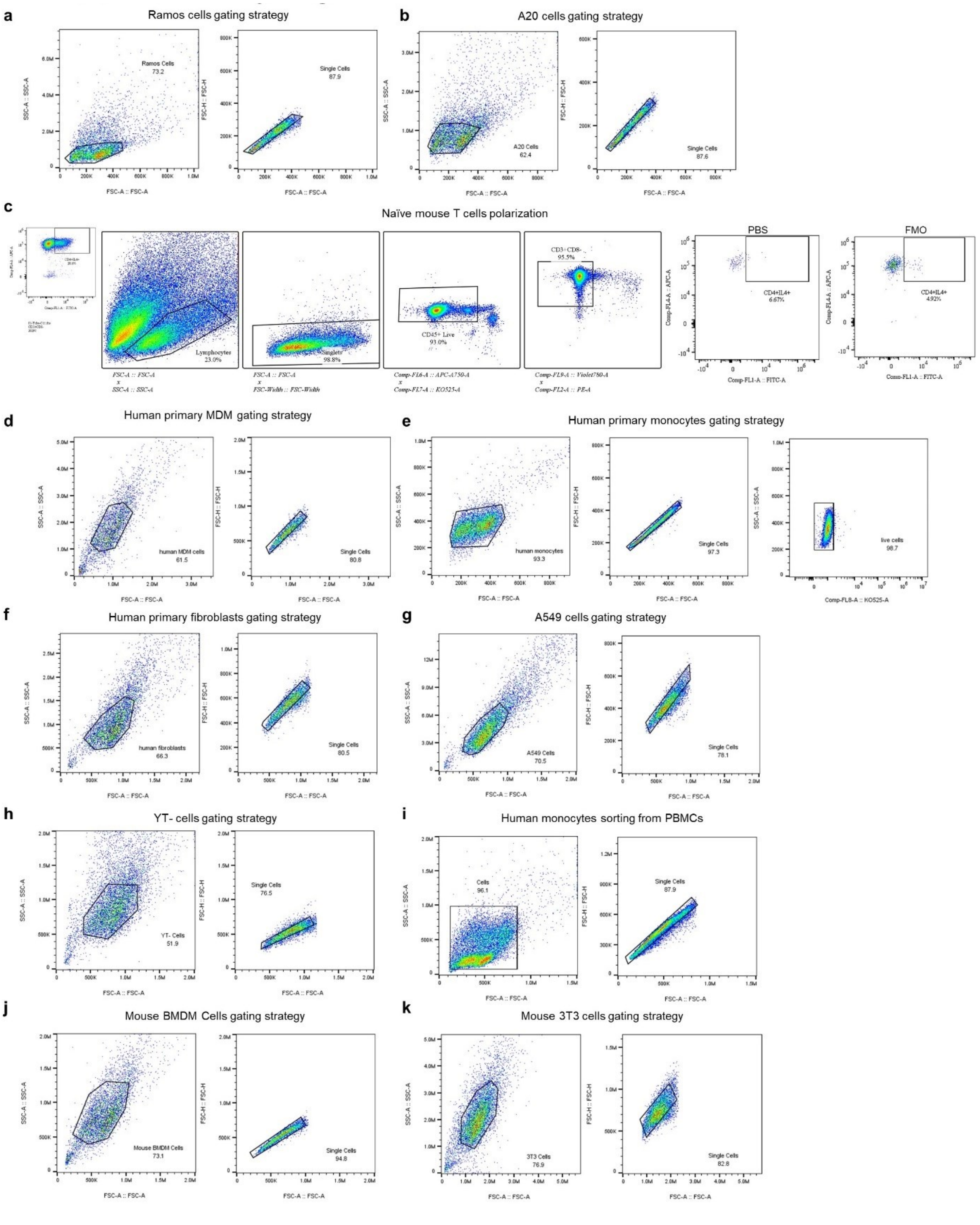

Supplementary Figure 1. Gating strategies used in all experiments with flow cytometry. a, Gating strategy for Ramos cells shown in **Fig. 2a, Fig. 5a** and **e, Fig 6a** and **f, Extended Data Fig. 4a, Extended Data Fig. 7c-d, Extended Data Fig. 8a,** and **Extended Data Fig. 9f** and **k. b,** Gating strategy for mouse A20 cells shown in **Fig. 2b, Extended Data Fig. 4b,** and **Extended Data Fig. 7b. c,** Gating strategy for

naïve mouse T cell polarization assay shown in **Fig. 2e-f. d,** Gating strategy for human primary MDM cells shown in **Fig. 5a, Extended Data Fig. 4a, Extended Data Fig. 7e-g,** and **Extended Data Fig. 8i. e,** Gating strategy for human primary monocytes shown in **Fig. 5a** and **f, Extended Data Fig. 4f** and **i,** and **Extended Data Fig. 7h-j. f,** Gating strategy for human primary fibroblasts shown in **Fig. 5a,** and **Extended Data Fig. 8i. g,** Gating strategy for human A549 cells shown in **Fig. 5a**, **Extended Data Fig. 7k-l** and **Extended Data Fig. 8b** and **i. h,** Gating strategy for human YT- cells shown in **Extended Data Fig. 4c. i,** Gating strategy for human primary monocytes sorting from PBMCs shown in **Extended Data Fig. 4g. j,** Gating strategy for mouse BMDM cells shown in **Fig. 6b, Extended Data Fig. 4b** and **j,** and **Extended Data Fig. 7b. k,** Gating strategy for mouse 3T3 cells shown in **Extended Data Fig. 7b.**

Supplementary Table 1 Correlated amino acids in human natural cytokine and de novo designed protein mimetics.

**hIL-4**	**Neo-2**	**Grafted hNeo-4**	**hNeo-4**
I5	L7	I7	
T6	H8	T8	M8
E9	H11	E11	
K12	Y14	K14	
S16	M18	S18	
	K68	K68	I68
Q78	K33	Q33	
R81	D36	R36	
F82	Y37	F37	
K84	F39	K39	
R85	N40	R40	
R88	L43	R43	
N89	I44	N44	
W91	E46	W46	
G92	E47	G47	
	I98	I98	F98

Supplementary Table 2 Correlated amino acids in mouse natural cytokine and de novo designed protein mimetics.

**mIL-4**	**Grafted hNeo-4**	**Grafted mNeo-4**	**mNeo-4**
H9	T8	H8	
G15	K14	G14	
I16	D15	I15	
E19	S18	E18	
S73	Q33	S33	
M76	R36	M36	
E77	F37	E37	
Q79	K39	Q39	
F82	E42	F42	I42
A84	N44	A44	
R86	W46	R46	G46
C87	G47	A47	G47
	A87	A87	L87
	I98	I98	N98
	F99	F99	Y99

Supplementary Table 3 Concentrations of soluble receptor chain(s) and sort statistics for selections of de novo designed protein mimetics. Biotinylated receptors are underlined, and biotinylated receptors that were tetramerized via incubation with streptavidin prior to labeling are *italicized*.

**Human IL-4 Mimic**	**hIL-4Rα**	**hγ_c**	**Number of sorted yeast cells**	**Number of selected yeast cells**
Sort 1	10 nM	-	100,000,000	50,000
Sort 2	750 pM	-	4,240,000	5,000
Sort 3	3 nM	50 nM	2,000,000	24,000
Sort 4	500 pM	1 nM	14,900,000	3,400
**Murine IL-4 Mimic**	**mIL-4Rα**	**mγ_c**	**Number of sorted yeast cells**	**Number of selected yeast cells**
Sort 1	*1 μM*	-	50,000,000	3,600
Sort 2	*500 nM*	-	6,860,000	100,000
Sort 3	100 nM	*100 nM*	1,140,000	8,800

Supplementary Table 4 Amino acid sequences of hNeo-4 and mNeo-4.

Name	Sequence
hNeo-4	PKKKIQIMAEEALKDALSILNIVKTNSPPAEEQLERFAKRFERNLWGIARLFESGD QKDEAEKAKRMIEWMKRIKTTASEDEQEEMANAIITILQSWFFS
mNeo-4	PKKKIQIHAEEALGIALEILNIVKTNSPPAEESLEMEAQRFIRALGGIARLFESGDQK DEAEKAKRMKEWMKRIKTTASEDEQEEMLNAIITILQSWNYS

Supplementary Table 5. Protein binding kinetic and equilibrium parameters.

	**Receptor chain**	**Kinetic fit values**					**Equilibrium EC50 (nM)**
		**K_D (nM)**	**k_{on} ($\times10^5$ 1/Ms)**	**k_{off} ($\times10^{-3}$ 1/s)**	**Full X^2**	**Full R^2**	
hIL-4	hIL-4Rα	0.478±0.003	5.92±0.02	0.283±0.001	1.94	0.985	1.68±0.07
	hIL-4Rα +hγ_c	64.0±0.5	1.15±0.01	7.33±0.03	0.202	0.990	48.1±3.7
	hIL-4Rα +hIL-13Rα	101±1	0.275±0.003	2.78±0.016	0.302	0.962	125±21
hNeo-4	hIL-4Rα	57.5±0.5	1.38±0.01	7.97±0.05	0.632	0.986	63.7±5.6

	hIL-4Rα +hγ_c	80.1±0.9	0.906±0.008	7.25±0.04	0.517	0.980	65.8±4.6
	hIL-4Rα +hIL-13Rα	ND	ND	ND	ND	ND	ND
	hIL-2Rβ	ND	ND	ND	ND	ND	ND
	hIL-2Rβ + hγ_c	ND	ND	ND	ND	ND	ND
Neo-2	hIL-2Rβ	24.8±0.2	2.14±0.02	5.30±0.03	0.381	0.985	12.3±1.4
	hIL-2Rβ + hγ_c	2.06±0.01	1.29±0.00	0.266±0.002	1.37	0.992	5.90±0.34
	hIL-4Rα	ND	ND	ND	ND	ND	ND
mIL-4	mIL-4Rα	3.88±0.07	5.70±0.06	2.21±0.03	0.952	0.954	3.25±0.51
	mIL-4Rα +mγ_c	ND	ND	ND	ND	ND	ND
mNeo-4	mIL-4Rα	77.9±2.5	19.7±0.5	154±3	0.277	0.983	71.2±4.1
	mIL-4Rα +mγ_c	ND	ND	ND	ND	ND	ND

Supplementary Table 6 EC_{50} values of treatments in signaling assays.

**Figure Number**	**Treatment**	**EC_{50} (M)**
Fig. 2a	hIL-4	1E-11
	hNeo-4	9.6E-11
Fig. 2c	mIL-4	1.38E-11
	mNeo-4	2.29E-08
Fig. 2f	mIL-4	1.72E-10
	mNeo-4	4.28E-09
Fig. 5a	hIL-4 (Ramos)	1.96E-11
	hIL-13 (Ramos)	ND
	hNeo-4 (Ramos)	2.86E-10
	hIL-4 (MDM)	2.94E-11
	hIL-13 (MDM)	2.36E-10
	hNeo-4 (MDM)	3.17E-09
	hIL-4 (Monocytes)	1.21E-11
	hIL-13 (Monocytes)	1.23E-10
	hNeo-4 (Monocytes)	1.53E-09
	hIL-4 (Fibroblasts)	2.89E-11
	hIL-13 (Fibroblasts)	4.11E-10
	hNeo-4 (Fibroblasts)	ND
	hIL-4 (A549)	6.37E-11
	hIL-13 (A549)	6.77E-10
	hNeo-4 (A549)	ND
Fig. 6a	hIL-4 (unheated)	1.71E-11
	hIL-4 (15 min)	3.89E-11

	hIL-4 (30 min)	1.07E-10
	hIL-4 (1 h)	7.02E-10
	hIL-4 (2 h)	2.25E-09
	hIL-4 (3 h)	7.43E-09
	hNeo-4 (unheated)	3.22E-10
	hNeo-4 (15 min)	1.72E-10
	hNeo-4 (30 min)	1.79E-10
	hNeo-4 (1 h)	3.19E-10
	hNeo-4 (2 h)	2.11E-10
	hNeo-4 (3 h)	2.25E-10
Fig. 6b	mIL-4 (unheated)	2.02E-11
	mIL-4 (1 h)	2.08E-08
	mNeo-4 (unheated)	1.87E-08
	mNeo-4 (1 h)	1.96E-08
Fig. 6f	hIL-4 (untreated)	6.67E-12
	hIL-4 (10 min)	1.53E-10
	hIL-4 (30 min)	1.84E-09
	hIL-4 (1 h)	6.60E-08
	hIL-4 (2 h)	ND
	hIL-4 (3 h)	ND
	hNeo-4 (untreated)	8.55E-11
	hNeo-4 (10 min)	3.30E-10
	hNeo-4 (30 min)	5.19E-09
	hNeo-4 (1 h)	4.62E-08
	hNeo-4 (2 h)	3.33E-08
	hNeo-4 (3 h)	2.79E-07
Extended Data Fig. 4a	hNeo-4 (Ramos)	1.28E-10
	Neo-2 (Ramos)	ND
	hIL-4 (MDM)	2.01E-11
	hNeo-4 (MDM)	3.28E-09
	Neo-2 (MDM)	ND
Extended Data Fig. 4b	mNeo-4 (A20)	1.61E-07
	Neo-2 (A20)	ND
	mIL-4 (BMDM)	9.49E-12
	mNeo-4 (BMDM)	6.73E-09
	Neo-2 (BMDM)	1.03E-06
Extended Data Fig. 4c	hIL-4	ND
	hNeo-4	ND
	Neo-2	5.69E-11
Extended Data Fig. 4f	hIL-4	6.81E-12
	hIL-13	5.56E-11
	hNeo-4	8.24E-10
Extended Data Fig. 4h	hIL-4 ($CD4^+$ T cells)	5.36E-12

	hIL-13 (CD4+ T cells)	ND
	hNeo-4 (CD4+ T cells)	4.51E-10
Extended Data Fig. 4i	hIL-4 (STAT1, Monocytes)	7.16E-11
	hIL-13 (STAT1, Monocytes)	1.28E-09
	hNeo-4 (STAT1, Monocytes)	4.33E-08
	hIL-4 (STAT3, Monocytes)	2.47E-10
	hIL-13 (STAT3, Monocytes)	1.71E-09
	hNeo-4 (STAT3, Monocytes)	1.12E-07
	hIL-4 (STAT5, Monocytes)	5.62E-11
	hIL-13 (STAT5, Monocytes)	1.19E-09
	hNeo-4 (STAT5, Monocytes)	9.31E-09
	hIL-4 (STAT6, Monocytes)	2.43E-12
	hIL-13 (STAT6, Monocytes)	5.26E-11
	hNeo-4 (STAT6, Monocytes)	3.36E-09
Extended Data Fig. 4i	mIL-4 (STAT1, BMDM)	2.41E-10
	mNeo-4 (STAT1, BMDM)	4.51E-07
	mIL-4 (STAT3, BMDM)	1.75E-10
	mNeo-4 (STAT3, BMDM)	2.18E-07
	mIL-4 (STAT5, BMDM)	1.06E-10
	mNeo-4 (STAT5, BMDM)	1.96E-07
	mIL-4 (STAT6, BMDM)	2.75E-11
	mNeo-4 (STAT6, BMDM)	3.51E-08
Extended Data Fig. 7b	mIL-4 (A20)	1.46E-10
	mNeo-4 (A20)	1.7E-07
	mIL-4 (Primary BMDMs Mouse 1)	4.37E-11
	mNeo-4 (Primary BMDMs Mouse 1)	3.56E-08
	mIL-4 (Primary BMDMs Mouse 2)	9.89E-12
	mNeo-4 (Primary BMDMs Mouse 2)	1.09E-08
	mIL-4 (3T3)	3.17E-10
	mNeo-4 (3T3)	ND
Extended Data Fig. 7c	hIL-4	1.96E-11
	hIL-4 with anti-IL-13R ab	2.07E-11
	hNeo-4	2.86E-10
	hNeo-4 with anti-IL-13R ab	3.49E-10
Extended Data Fig. 7d	hIL-4	2.94E-11
	hIL-4 with anti-IL-13R ab	1.31E-10
	hIL-13	2.36E-10
	hIL-13 with anti-IL-13R ab	5.84E-09
	hNeo-4	3.17E-09
	hNeo-4 with anti-IL-13R ab	2.2E-09
Extended Data Fig. 7e	hIL-4	1.21E-11
	hIL-4 with anti-IL-13R ab	4.67E-11
	hIL-13	1.23E-10

	hIL-13 with anti-IL-13R ab	2.15E-09
	hNeo-4	1.53E-09
	hNeo-4 with anti-IL-13R ab	2.18E-09
Extended Data Fig. 7f	hIL-4	3.73E-11
	hIL-4 with anti-IL-13R ab	2.01E-10
	hIL-13	6.52E-10
	hIL-13 with anti-IL-13R ab	4.94E-09
Extended Data Fig. 8a	hIL-4	8.45E-12
	hNeo-4	9.1E-11
	super-4	3.09E-11
Extended Data Fig. 8b	hIL-4	7.88E-11
	hNeo-4	ND
	super-4	1.07E-09
Extended Data Fig. 9f	hIL-4	1.08E-11
	hNeo-4	1.44E-10
	hNeo-4 (lyophilized)	1.72E-10
Extended Data Fig. 9k	Soluble hNeo-4	1.75E-10
	hNeo-4 M	3.14E-10
	hNeo-4 S	6.37E-08
	PCL M	2.06E-10
	PCL S	9.29E-09

Supplementary Table 7 Statistical significance for all applicable figure panels. p values smaller than 0.000000001 are indicated as <1E-9.

Figure	Statistical Test	Comparison	Significant ?	Summary	Adjusted P Value
Fig. 2c	Tukey's multiple comparisons test	PBS vs. hIL-4	Yes	****	0.00000081
		PBS vs. hNeo-4	Yes	****	0.000001
		hIL-4 vs. hNeo-4	No	ns	0.9414169
Fig. 2d	Tukey's multiple comparisons test	PBS vs. mIL-4	Yes	****	0.000000002
		PBS vs. mNeo-4	Yes	****	0.000000001
		mIL-4 vs. mNeo-4	Yes	*	0.017942723
Fig. 4b and Extended Data Fig. 6e	Tukey's multiple comparisons test	*Arg1*			
		PBS vs. mIL-4	Yes	****	0.00000341

		PBS vs. mNeo-4 High	Yes	****	0.000000152
		PBS vs. mNeo-4 Medium	No	ns	0.131063332
		PBS vs. mNeo-4 Low	No	ns	0.404279827
		mIL-4 vs. mNeo-4 High	No	ns	0.806894502
		mIL-4 vs. mNeo-4 Medium	Yes	*	0.035473905
		mIL-4 vs. mNeo-4 Low	Yes	****	0.00000001
		mNeo-4 High vs. mNeo-4 Medium	Yes	**	0.00256164
		mNeo-4 High vs. mNeo-4 Low	Yes	****	<1E-9
		mNeo-4 Medium vs. mNeo-4 Low	Yes	**	0.001790302
		Chil3			
		PBS vs. mIL-4	Yes	****	0.000000071
		PBS vs. mNeo-4 High	Yes	****	<1E-9
		PBS vs. mNeo-4 Medium	No	ns	0.909891843
		PBS vs. mNeo-4 Low	No	ns	0.997848608
		mIL-4 vs. mNeo-4 High	No	ns	0.521946586
		mIL-4 vs. mNeo-4 Medium	Yes	****	0.000010093
		mIL-4 vs. mNeo-4 Low	Yes	****	0.000001169
		mNeo-4 High vs. mNeo-4 Medium	Yes	****	0.000000079
		mNeo-4 High vs. mNeo-4 Low	Yes	****	0.000000009
		mNeo-4 Medium vs. mNeo-4 Low	No	ns	0.98509873
		Ccl24			
		PBS vs. mIL-4	Yes	****	<1E-9
		PBS vs. mNeo-4 High	Yes	****	<1E-9
		PBS vs. mNeo-4 Medium	No	ns	0.38812475
		PBS vs. mNeo-4 Low	No	ns	0.999999851
		mIL-4 vs. mNeo-4 High	No	ns	0.746453211
		mIL-4 vs. mNeo-4 Medium	Yes	****	0.000005233
		mIL-4 vs. mNeo-4 Low	Yes	****	0.000000004

		mNeo-4 High vs. mNeo-4 Medium	Yes	****	0.000000166
		mNeo-4 High vs. mNeo-4 Low	Yes	****	<1E-9
		mNeo-4 Medium vs. mNeo-4 Low	No	ns	0.458637045
		Retnla			
		PBS vs. mIL-4	Yes	****	<1E-9
		PBS vs. mNeo-4 High	Yes	****	<1E-9
		PBS vs. mNeo-4 Medium	Yes	*	0.027532877
		PBS vs. mNeo-4 Low	No	ns	0.977981539
		mIL-4 vs. mNeo-4 High	No	ns	0.765575285
		mIL-4 vs. mNeo-4 Medium	Yes	****	0.000011109
		mIL-4 vs. mNeo-4 Low	Yes	****	<1E-9
		mNeo-4 High vs. mNeo-4 Medium	Yes	****	0.000000398
		mNeo-4 High vs. mNeo-4 Low	Yes	****	<1E-9
		mNeo-4 Medium vs. mNeo-4 Low	No	ns	0.148734967
Fig. 4c and Extended Data Fig. 6f	Tukey's multiple comparisons test	*Arg1*			
		PBS vs. mIL-4	Yes	****	0.000015806
		PBS vs. mNeo-4 Low	Yes	*	0.018377913
		PBS vs. mNeo-4 Med	Yes	****	0.000017069
		PBS vs. mNeo-4 High	Yes	****	0.000000616
		mIL-4 vs. mNeo-4 Low	No	ns	0.259223923
		mIL-4 vs. mNeo-4 Med	No	ns	0.999999966
		mIL-4 vs. mNeo-4 High	No	ns	0.80786661
		mNeo-4 Low vs. mNeo-4 Med	No	ns	0.268286278
		mNeo-4 Low vs. mNeo-4 High	Yes	*	0.027920202
		mNeo-4 Med vs. mNeo-4 High	No	ns	0.798269422
		Chil3			
		PBS vs. mIL-4	No	ns	0.607134377

		PBS vs. mNeo-4 Low	No	ns	0.999954568
		PBS vs. mNeo-4 Med	No	ns	0.721580539
		PBS vs. mNeo-4 High	No	ns	0.968421687
		mIL-4 vs. mNeo-4 Low	No	ns	0.681692403
		mIL-4 vs. mNeo-4 Med	No	ns	0.999736569
		mIL-4 vs. mNeo-4 High	No	ns	0.95230939
		mNeo-4 Low vs. mNeo-4 Med	No	ns	0.788811039
		mNeo-4 Low vs. mNeo-4 High	No	ns	0.984448652
		mNeo-4 Med vs. mNeo-4 High	No	ns	0.982607689
		Ccl24			
		PBS vs. mIL-4	Yes	****	0.000006155
		PBS vs. mNeo-4 Low	No	ns	0.92673559
		PBS vs. mNeo-4 Med	No	ns	0.398924134
		PBS vs. mNeo-4 High	Yes	****	0.000003389
		mIL-4 vs. mNeo-4 Low	Yes	***	0.000142334
		mIL-4 vs. mNeo-4 Med	Yes	**	0.003996731
		mIL-4 vs. mNeo-4 High	No	ns	0.990705035
		mNeo-4 Low vs. mNeo-4 Med	No	ns	0.872439141
		mNeo-4 Low vs. mNeo-4 High	Yes	****	0.000068622
		mNeo-4 Med vs. mNeo-4 High	Yes	**	0.001766211
		Retnla			
		PBS vs. mIL-4	Yes	*	0.01903802
		PBS vs. mNeo-4 Low	No	ns	0.063121193
		PBS vs. mNeo-4 Med	Yes	**	0.002248871
		PBS vs. mNeo-4 High	Yes	**	0.00863488
		mIL-4 vs. mNeo-4 Low	No	ns	0.991182606
		mIL-4 vs. mNeo-4 Med	No	ns	0.957565978
		mIL-4 vs. mNeo-4 High	No	ns	0.991400691
		mNeo-4 Low vs. mNeo-4 Med	No	ns	0.782218667

		mNeo-4 Low vs. mNeo-4 High	No	ns	0.904855151
		mNeo-4 Med vs. mNeo-4 High	No	ns	0.999610702
Extended Data Fig. 4d	Tukey's multiple comparisons test	PBS vs. hIL-4	Yes	**	0.0011
		PBS vs. hNeo-4	Yes	***	0.0008
		hIL-4 vs. hNeo-4	No	ns	0.9576
Extended Data Fig. 4e	Tukey's multiple comparisons test	*Chil3*			
		PBS vs. mIL-4	Yes	****	<1E-9
		PBS vs. mNeo-4	Yes	****	<1E-9
		mIL-4 vs. mNeo-4	No	ns	0.271785481
		Il1b			
		PBS vs. mIL-4	Yes	****	<1E-9
		PBS vs. mNeo-4	Yes	****	0.000000005
		mIL-4 vs. mNeo-4	Yes	*	0.02935627
Extended Data Fig. 6c	Sidak's multiple comparisons test	*Arg1*	Yes	****	0.000008291
		Chil3	Yes	****	0.000006681
		Ccl24	Yes	****	0.000000272
		Retnla	Yes	****	0.000000148
Extended Data Fig. 6d	Tukey's multiple comparisons test	*Arg1*			
		PBS vs. mIL-4	Yes	****	<1E-9
		PBS vs. mNeo-4	Yes	****	0.000002999
		mIL-4 vs. mNeo-4	Yes	****	0.000085998
		Chil3			
		PBS vs. mIL-4	Yes	****	<1E-9
		PBS vs. mNeo-4	Yes	****	0.000073812
		mIL-4 vs. mNeo-4	Yes	****	0.000000007
		Ccl24			
		PBS vs. mIL-4	Yes	****	<1E-9
		PBS vs. mNeo-4	No	ns	0.094004708
		mIL-4 vs. mNeo-4	Yes	****	<1E-9
		Retnla			
		PBS vs. mIL-4	Yes	****	<1E-9
		PBS vs. mNeo-4	No	ns	0.993538532

		mIL-4 vs. mNeo-4	Yes	****	<1E-9

Supplementary Table 8 Human IL-4 related receptor quantification on various cell lines.

Cell type	Donor/ test #	Receptor	Number of receptors	γc:IL-13Rα	IL-13Rα:IL-4Rα	γc:IL-4Rα
human monocytes	Donor 1	IL-4Rα	129	184	5.96	1100
		γ_c	141226			
		IL-13Rα	766			
	Donor 2	IL-4Rα	81	418	6.73	2810
		γ_c	227469			
		IL-13Rα	545			
	Donor 3	IL-4Rα	112	274	4.37	1200
		γ_c	133560			
		IL-13Rα	487			
	Donor 4	IL-4Rα	169	89.2	4.00	357
		γ_c	60489			
		IL-13Rα	678			
	Donor 5	IL-4Rα	184	229	2.99	685
		γ_c	125764			
		IL-13α	550			
	Donor 6	IL-4Rα	50	108	10.0	1080
		γ_c	53644			
		IL-13Rα	495			
human macrophage	Donor 1	IL-4Rα	ND	381	ND	ND
		γ_c	843885			
		IL-13Rα	2214			
	Donor 2	IL-4Rα	ND	462	ND	ND
		γ_c	778759			
		IL-13Rα	1686			
	Donor 3	IL-4Rα	ND	325	ND	ND
		γ_c	1686134			
		IL-13Rα	5190			
	Donor 4	IL-4Rα	ND	434	ND	ND
		γ_c	960148			
		IL-13Rα	2214			
human fibroblasts	Test 1	IL-4Rα	695	85.6	1.39	119
		γ_c	82826			
		IL-13Rα	968			
	Test 2	IL-4Rα	842	120	0.304	36.5
		γ_c	30706			
		IL-13Rα	256			
	Test 3	IL-4Rα	552	13.6	3.13	42.5
		γ_c	23,469			
		IL-13Rα	1,730			
Ramos	Test 1	IL-4Rα	3120	ND	ND	6.67
		γ_c	20818			

		IL-13Rα	ND			
	Test 2	IL-4Rα	2887	2550	0.00	4.43
		γ_c	12789			
		IL-13Rα	5			
A549	Test 1	IL-4Rα	110	6.02	13.7	82.5
		γ_c	9086			
		IL-13Rα	1510			
	Test 2	IL-4Rα	143	5.42	9.91	53.7
		γ_c	7663			
		IL-13Rα	1413			

Supplementary Table 9 Mouse IL-4 related receptor quantification on various cell lines.

Cell type	Donor/ test #	Receptor	Number of receptors	γ_c:IL-13Rα	IL-13Rα:IL-4Rα	γ_c:IL-4Rα
mouse primary macrophages	Mouse 1	IL-4Rα	33534	0.886	1.02	0.902
		γ_c	30256			
		IL-13Rα	34140			
	Mouse 2	IL-4Rα	23600	1.45	0.666	0.965
		γ_c	22764			
		IL-13Rα	15708			
3T3	Test 1	IL-4Rα	7095	0.288	2.27	0.654
		γ_c	4642			
		IL-13Rα	16119			
A20	Test 1	IL-4Rα	1948	2.39	0.453	1.09
		γ_c	2114			
		IL-13Rα	883			

Supplementary Table 10 hNeo-4 crystallography Data collection and refinement statistics. Statistics for the highest-resolution shell are shown in parentheses.

	hNeo-4
Wavelength	1.033
Resolution range	40.91 - 2.972 (3.078 - 2.972)
Space group	R 3 2 :H
Unit cell (Å, °)	122.477 122.477 310.376 90 90 120
Total reflections	187460 (18676)
Unique reflections	18828 (1823)
Multiplicity	10.0 (10.2)
Completeness (%)	99.76 (98.91)
Mean I/sigma(I)	7.79 (0.72)
R-merge	0.1516 (3.134)
R-meas	0.1602 (3.3)
R-pim	0.05144 (1.027)
CC1/2	0.999 (0.258)

Reflections used in refinement	18817 (1820)
Reflections used for R-free	935 (90)
R-work	0.2213 (0.3429)
R-free	0.2601 (0.3947)
Number of non-hydrogen atoms	5991
macromolecules	5991
ligands	0
solvent	0
Protein residues	765
RMS(bonds)	0.004
RMS(angles)	0.65
Ramachandran favored (%)	97.56
Ramachandran allowed (%)	2.44
Ramachandran outliers (%)	0.00
Rotamer outliers (%)	4.78
Clashscore	8.46
Average B-factor	126.58
macromolecules	126.58
Number of TLS groups	8

Supplementary Table 11 Taqman probes used for qRT-PCR evaluations.

Species	Probe	Assay ID:
Human	*GAPDH*	Hs02786624_g1
	CD209	Hs01588349_m1
	CD200R1	Hs00793597_m1
	TLR8	Hs00152972_m1
	CTSK	Hs00166156_m1
	ALOX15	Hs00609608_m1
	CISH	Hs01003603_m1
	MRC1	Hs00267207_m1
	SPINT2	Hs01070442_m1
Mouse	*Hprt*	Mm03024075_m1
	Rer1	Mm00471276_m1
	Arg1	Mm00475988_m1
	Chil3	Mm00657889_mH
	IL1B	Mm00434228_m1
	Ym1	Mm00657889_mH
	Retnla	Mm00445109_m1
	Ccl24	Mm00444701_m1

Supplementary Table 12 Dilution volumes for the signaling assay validating bioactivity of 3D-printed scaffolds.

Number of Concentration	Released hNeo-4 samples			hNeo-4 scaffold samples		
	Supernatant volume from overnight incubation added (µL)	Additional fresh media added (µL)	Final concentration (M)	Supernatant volume from overnight incubation added (µL)	Additional fresh media added (µL)	Final concentration (M)
1	25	0	4.41E-09	0	25.0	6.59E-07
2	25	87.5	9.81E-10	0	112.5	1.46E-07
3	25	481.3	2.18E-10	0	506.3	3.25E-08
4	25	2253.1	4.84E-11	0	2278.1	7.23E-09
5	25	10226.6	1.08E-11	0	10251.6	1.61E-09
6	25	46107.0	2.39E-12	0	46132.0	3.57E-10
