# Supplementary figures and images for: Design of cell-type-specific hyperstable IL-4 mimetics via modular de novo scaffolds
Source: Nat Chem Biol. Author manuscript; Available in PMC 2023 Dec 5. (PMC10697138; doi:10.1038/s41589-023-01313-6)

# Extended Data Figure 1

**e**

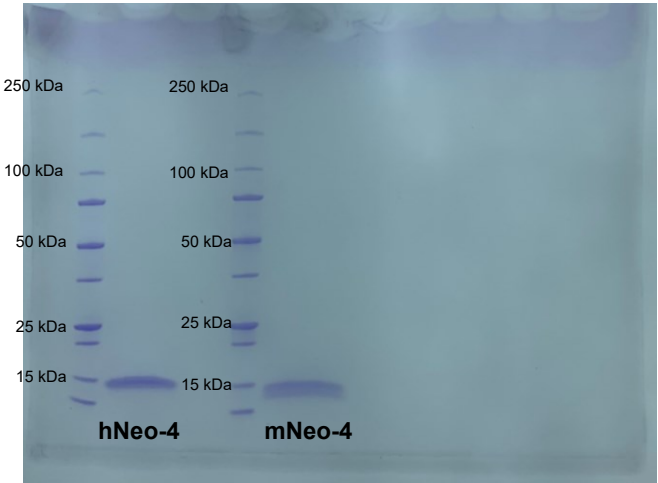

Supplement: source data extended data fig 1 [file NIHMS1917493-supplement-source_data_extended_data_fig_1.pdf]
